# DALib: A Curated Repository of Libraries for Data Augmentation in Computer Vision

**DOI:** 10.3390/jimaging9100232

**Published:** 2023-10-20

**Authors:** Sofia Amarù, Davide Marelli, Gianluigi Ciocca, Raimondo Schettini

**Affiliations:** Department of Informatics, Systems and Communication, University of Milano-Bicocca, Viale Sarca 336, 20126 Milano, Italy; s.amaru@campus.unimib.it (S.A.); gianluigi.ciocca@unimib.it (G.C.); raimondo.schettini@unimib.it (R.S.)

**Keywords:** data augmentation, deep learning, computer vision, libraries

## Abstract

Data augmentation is a fundamental technique in machine learning that plays a crucial role in expanding the size of training datasets. By applying various transformations or modifications to existing data, data augmentation enhances the generalization and robustness of machine learning models. In recent years, the development of several libraries has simplified the utilization of diverse data augmentation strategies across different tasks. This paper focuses on the exploration of the most widely adopted libraries specifically designed for data augmentation in computer vision tasks. Here, we aim to provide a comprehensive survey of publicly available data augmentation libraries, facilitating practitioners to navigate these resources effectively. Through a curated taxonomy, we present an organized classification of the different approaches employed by these libraries, along with accompanying application examples. By examining the techniques of each library, practitioners can make informed decisions in selecting the most suitable augmentation techniques for their computer vision projects. To ensure the accessibility of this valuable information, a dedicated public website named DALib has been created. This website serves as a centralized repository where the taxonomy, methods, and examples associated with the surveyed data augmentation libraries can be explored. By offering this comprehensive resource, we aim to empower practitioners and contribute to the advancement of computer vision research and applications through effective utilization of data augmentation techniques.

## 1. Introduction

Data augmentation (DA) is a fundamental technique in machine learning that aims to artificially expand the size of the training dataset by applying various transformations or modifications to the existing data. By augmenting the data, we introduce new variations and increase the diversity of the training examples, leading to improved model performance, generalization, and robustness.

Deep-learning-based approaches are very data-intensive, thus researchers have devoted time and energy in creating larger and larger collections of data that are both representative and comprehensive for a given application domain. However, this is very time-consuming task and for some application domains, such large collection of data are very difficult to obtain.

For this reason, data augmentation has become a critical component in deep learning pipelines, especially when faced with limited labeled data, or where increasing the diversity and robustness of the dataset are crucial. A variety of tasks benefit from data augmentation; some examples are:

- Computer Vision—Data augmentation is extensively employed in computer vision tasks, such as image classification, object detection, and semantic segmentation. By applying transformations like rotations, translations, flips, zooms, and color variations, data augmentation helps models learn to recognize objects from different perspectives, lighting conditions, scales, and orientations [1,2,3,4]. This enhances the generalization ability of the models and makes them more robust to variations in real-world images.
- Natural Language Processing (NLP)—Data augmentation techniques are also applicable to NLP tasks, including text classification, sentiment analysis, and machine translation [5,6,7]. Methods like back-translation, word replacement, and synonym substitution can be used to generate augmented text samples, effectively increasing the diversity of the training data and improving the language model’s understanding and generalization.
- Speech Recognition—In speech recognition tasks, data augmentation techniques can be used to introduce variations in audio samples, such as adding background noise, altering pitch or speed, or applying reverberations [8,9,10]. Augmenting the training data with these transformations helps a model to be more robust to different acoustic environments and improves its performance in real-world scenarios.
- Time Series Analysis—Data augmentation can be applied to time series data, such as sensor data, stock market data, or physiological signals. Techniques like time warping, scaling, jittering, and random noise injection can create augmented samples that capture various temporal patterns, trends, and noise characteristics [11,12,13]. Augmentation in time series data helps models to learn and generalize better to different temporal variations and noisy conditions.
- Medical Imaging—In the field of medical imaging, data augmentation is crucial due to the scarcity and high cost of labeled data. Augmentation techniques such as rotations, translations, and elastic deformations can simulate anatomical variations, different imaging angles, and distortions in medical images [14,15,16,17]. By augmenting the dataset, models trained on limited labeled medical images can generalize better and be more robust to variability in patient anatomy and imaging conditions.
- Anomaly Detection—Data augmentation can be used to create artificial anomalies or perturbations in normal data samples, thereby generating augmented data that contains both normal and anomalous instances [18,19,20]. This augmented dataset can then be used for training anomaly detection models, enabling them to learn a wider range of normal and abnormal patterns and improving their detection accuracy.

**Figure 1:**
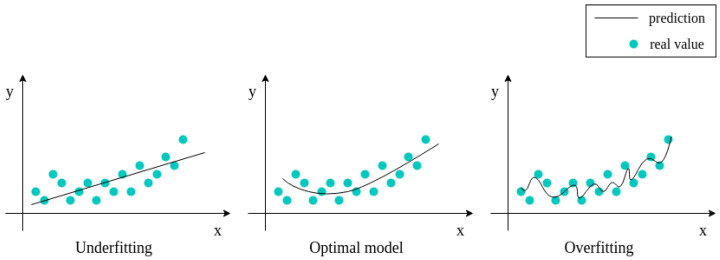
Graphical representation of underfitting and overfitting issues.

Data augmentation offers several benefits. It is an easy way to collect a more extensive and diverse dataset without the need of additional labeled samples. This improves the generalization capabilities of learned models due to variability introduced in the training. The models become more invariant to real-world data noise and common variations. By enlarging the amount of available data, data augmentation can effectively mitigate the risk of model overfitting when there are few samples. It can also resolve class imbalance issues by artificially balancing the representation of minority classes that causes underfitting (see Figure 1).

As stated before, data augmentation is very powerful but the techniques to be used vary depending on the application scenario. In the literature, there are many libraries designed to provide standard or ad hoc data augmentation strategies. Since each library may contains tens of such strategies, it is often difficult to identify which library could be more suitable for a given application scenario.

In this work, we want to provide a comprehensive survey of the publicly available data augmentation libraries to help practitioners to navigate through them by providing a curated taxonomy of the different approaches and application examples. The aim is not to identify the best data augmentation library but to offer an overview of their content allowing users to select the most-suitable data augmentation strategies among all the libraries for their problem. To this end, we have researched the available data augmentation libraries focusing on those pertaining to computer vision tasks. From each library, we extracted the implemented methods, categorized them, and composed a taxonomy of augmentation methods. We considered both generic methods, i.e., those that are common to and can be applied to different tasks, and task-specific methods. For each method, we compose a brief card describing its usage, expected results and parameters. All the gathered information is collected in a public website DALib: http://www.ivl.disco.unimib.it/activities/dalib/ (accessed on 10 October 2023) where the taxonomy, methods, and examples can be navigated and consulted. The contributions of our work are:

1. We collect information on data augmentation libraries designed for computer vision applications. Existing reviews focus on data augmentation methods and not on the availability of such methods in public libraries;
2. Our work is not limited to a specific application scenario. The libraries have been chosen to offer the readers a comprehensive survey of available methods to be used in different computer vision applications;
3. To the best of our knowledge, this is the first comprehensive review of data augmentation libraries that also offers a curated taxonomy;
4. We also provide a dedicated public website to serve as a centralized repository where the taxonomy, methods, and examples associated with the surveyed data augmentation can be explored;
5. This is an ongoing work that will be expanded when new libraries become available.

The remainder of this paper is structured as follows. Section 2 introduces the data augmentation libraries considered in this work. Section 3 defines a taxonomy of data augmentation techniques, organizing the methods available in the considered libraries. Section 4 describes the commonly used techniques for data augmentation in computer vision tasks. Section 5 presents DALib, a website that helps the user to discover the data augmentation techniques available in the libraries with visual and code examples. Section 6 illustrates some challenges and drawbacks of using data augmentation techniques. Finally, in Section 7, we report our conclusions.

## 2. Data Augmentation Libraries

Several libraries have been developed in recent years to simplify the use of different data augmentation strategies for several tasks. Here, we focus on the arguably most-used libraries for data augmentation in computer vision tasks. Although, data augmentation techniques can be implemented for specific tasks using low-level computer vision libraries (e.g., OpenCV, Pillow), creating custom implementations can be challenging and time consuming. The use of well-established libraries allows researchers to reduce the work burden and avoid errors in the coding of the methods. We also do not take into consideration implementations of single specific strategies or methods. After extensive research, 11 libraries have been identified (see Table 1): Albumentations [21], AugLy [22], Augmentor [23], Augraphy [24], Automold [25], CLoDSA [26], imgaug [27], KerasCV [28], Kornia [29], SOLT [30], and Torchvision [31]. The libraries have been selected based on their popularity, number of data augmentation methods implemented, and documentation availability and completeness. Most of these are general purpose with the exception of the domain-specific Augraphy and Automold libraries. The former is intended for the data augmentation of document images; the latter is specifically designed for the augmentation of road images. Although of general use, some of the libraries include domain-specific methods as well. We will delve deeper into the matter during the deeper investigation of the individual libraries.

Table 2 provides an overview of the libraries. All the libraries use Python as the programming language. They commonly provide integration with machine learning and deep learning frameworks such as PyTorch and Keras.

The libraries support a wide number of data augmentation techniques. Automold provide the lowest number with 17 (understandable since it is very task specific), while imgaug has more than 170 with general, broad-range, techniques. All the repositories have been updated in the last three years. Most of the libraries receive frequent updates. In Table 2, we also report some indices of library popularity. Specifically, we included the number of GitHub stars that each library received, the number of citations for the original paper describing the library (if available), and the number of works referencing the library in any form. For these latter two analyses, we use the search results from Google Scholar. Our research shows that the most popular data augmentation libraries are Albumentations, imgaug and Torchvision. It should be noted that Torchvision is the default data augmentation library for the PyTorch ML framework.

The following subsections provide further details of the libraries and their features.

### 2.1. Albumentations

Albumentations [21] is a Python library for image augmentation, first released in 2018, which boasts of being particularly fast and flexible. The library is part of the PyTorch ecosystem and can be integrated with deep learning frameworks like PyTorch and Keras. Image transformations fall in two macro-categories: pixel-based, which effect image contents, and spatial-based, which change image structure. Between the two categories, we have identified 83 image transformations (ignoring basic data type modifiers and support ones). The authors of the library adopted a set of design principles to provide a balanced approach that addresses current needs in data augmentation. These five principles are: Performance, which is guaranteed by using a vast number of dependencies in order to relay on the best-in-class implementation for each base transformation; Variety, to provide a vast, diverse, and combinable set of image transformations in order to support different use case scenarios; Conciseness, by providing a simple interface to the user and hiding the complexity of the transformations; Flexibility, to guide the development of the library by quickly adapting to the evolving needs of researchers and companies; and Open-source, to allow the easy reproduction of results and to build a solid code-base. By sticking to those principles, the authors provide a general-purpose library that implements the most-common image transformation operations as well as a vast set of generic and domain-specific augmentation techniques. It also includes methods from Automold to enhance street images. With Albumentations, it is also possible to increase masks, bounding-boxes, and key points (limited to the applied transformation).

The project also includes a demo website (https://demo.albumentations.ai/, accessed on 10 October 2023) which shows, in an immediate way, the functioning of a subset of the available transformations. The tool allows the user to upload their own image, modify the parameters through an intuitive user interface, and obtain the relative source code.

### 2.2. AugLy

AugLy [22] is an open-source DA library written in Python by Joanna Bitton and Zoe Papakipos, software engineers at Meta AI—an artificial intelligence lab of Meta Platforms (formerly named Facebook)—focused on user activity on social platforms such as Facebook and Instagram. The library only supports the PyTorch deep learning framework. AugLy combines several modes: audio, image, video, and text. Each mode constitutes a sublibrary. More than 100 augmentation methods are provided across the different modalities, 38 of which are image augmentations. The image data augmentation techniques are implemented in the augly.image package. This sublibrary contains, in addition to a wide range of generic techniques, specific methods (e.g., *MemeFormat*, *OverlayEmoji*, *OverlayOntoScreenshot*, *OverlayText*, etc.) that modify the input images in a similar way to how a user who has taken a screenshot, decorated a photo, or created a funny meme would. This type of DA increases data usefulness for training copy detection, hate speech detection, or copyright infringement models. In addition to the previous operations, AugLy supports the composition of multiple augmentations, applying them with a given probability; it also supports multimodal augmentations (e.g., frames and audio in a video) making it the first multimodal data augmentation library, according to the authors. The library was built with the robustness and the vast landscape of organic data augmentations seen online in mind.

### 2.3. Augmentor

Augmentor [23] is a generic image augmentation library. It aims to be a platform- and framework-independent standalone library to allow for more granular control over augmentations and implement augmentation techniques more relevant to the real world. Nevertheless, Pytorch, Keras, and Flux are out-of-the-box supported frameworks. Augmentor is written in Python but there is a sister project that uses the Julia programming language. The library uses a stochastic, pipeline-based approach that allows to chain augmentation operations together. All operations in the pipeline are applied stochastically, in terms of both the probability of the operations being applied and the value of each operation’s parameters. Thanks to the highly parametric operations of Augmentor, it is possible to achieve fine control over how images are created and generate realistically feasible training data. The image transformations within Augmentor are broadly categorized into six groups: perspective skewing, elastic distortions, rotation, shearing, cropping, and mirroring. In total the library contains 21 transformations.

### 2.4. Augraphy

Augraphy [24] is an image augmentation library written in Python that synthesizes images related to paper documents, scans, faxes, prints, and photocopies. The augmentation pipeline is highly configurable. It starts from a clean document by extracting text and graphics from the source to create an ink layer. After the creation of the ink layer, the augmentation pipeline proceeds to manipulate and deteriorate the text and graphics through distortion and degradation techniques. At the same time, it is possible to generate a paper texture layer, which, in turn, can be processed via a pipeline. The two layers are combined, resulting in an image that can be further enhanced by introducing creases, distortions, or other effects. The image transformations comprise standard transformations operating at pixel level, and deterioration of the document appearance by incorporating visual distortions such as glitches, ink-related issues, scribbles, and cast shadows, among others. In total, we have identified 26/40 image transformations in the library. The library does not directly support specific machine learning frameworks; however, it works with NumPy arrays that can be easily converted to formats used by those frameworks.

### 2.5. Automold

Self-driving cars have to overcome several challenges: being able to drive in blinding light conditions, at night, on foggy days, on snow-covered roads, recognizing potential obstacles to avoid, and maintaining a low speed if the road is wet or covered with gravel. There are many such scenarios and in order to train a CNN adequately, it is necessary to either have a large dataset that contains all the scenarios, or to remedy the problem via DA. In 2018, Ujjwal Saxena [25] set out to find a DA library to synthesize road images in a specific way and not finding valid options, he decided to create one. Thus was born Automold [25], a DA library dedicated to augmenting road images.

Automold is written in Python and uses the OpenCV [32] and NumPy packages. The library contains some generic methods to change brightness, correct exposure, or flip the image. The library also includes very specific augmentations techniques tailored for road image analysis. Among these techniques we can list: add_autumn, add_fog, add_gravel, add_manhole, add_rain, add_shadow, add_snow, add_speed, and add_sun_flare. All these augmentations can be exploited in automotive applications to design algorithms robust to different environment conditions. Automold contains a total of 17 augmentation techniques.

### 2.6. CLoDSA

CLoDSA [26] is a Python library designed for augmenting data in computer vision tasks, including object classification, localization, tracking, semantic segmentation, and instance segmentation. It offers extensive augmentation options and allows the user to easily combine them. In addition to 2D images, CLoDSA also supports collection of images such as stacks of images or videos. The augmentations supported by CLoDSA are fairly standard, ranging from color-based transformations and filtering to geometric and spatial variations for a total of 26 transformations. However, one of the key features of the library lies in the way it handles the annotations associated with the input data. Specifically, CLoDSA can directly use the annotation files generated from various annotation tools and applications. Among the supported annotation formats are: COCO, Pascal VOC, YOLO, ImageJ, and Youtube BB. These can be fed to deep learning algorithms without the need of a pre-processing step.

### 2.7. imgaug

imgaug [27] is a popular open-source image augmentation library written in Python. It provides extensive support for various augmentation techniques, enabling effortless combination and execution in random order or across multiple CPU cores. In addition to image augmentation, it also supports augmentation of key points/landmarks, bounding boxes, heat maps, and segmentation maps. It includes a variety of augments, including some aimed at altering atmospheric conditions adding rain, cloud cover, snowfall, and fog. Among all the augmentation libraries, imgaug is the largest and most-comprehensive. The augmentation techniques are organized into 16 groups: Arithmetic, Artistic, Blend, Blur, Color, Contrast, Convolutional, Edges, Flip, Geometric, ImgCorruptLIke, PillLike, Pooling, Segmentation, Size, and Weather. Each category contains several approaches. For example, the blur category contains six different strategies for applying the blur effect: Gaussian, Median, Average, Bilateral, Motion Blur and Mean Shift. In total, imgaug supports 164 augmentation techniques.

### 2.8. KerasCV

Keras is a Python-based deep learning API that operates on the TensorFlow machine learning platform. KerasCV [28] is a library of modular computer-vision-oriented Keras components including models, layers, metrics, loss functions, callbacks, and other utility functions. The subclass of keras.layers.Layer includes Augmentation and Preprocessing layers, which encompass standard image augmentation transformations that are commonly used. KerasCV offers a helpful base class (*BaseImageAugmentationLayer*) for writing custom data augmentation layers. Moreover, it also provides functionalities to easily generate novel images based on text prompts using text-to-image models. Specifically, KerasCV implements Stable Diffusion [33] optimized to achieve fast generation speed (not included in the augmentation library). KerasCV contains 21 augmentations covering all the standard image augmentation approaches.

### 2.9. Kornia

Kornia [29] is a computer vision library written in Python and based on PyTorch. It consists of a set of routines and differentiable modules for solving generic computer vision problems. This library offers a wide range of image processing functions and operators, such as geometric manipulation, image enhancement, tensor transformation, and coordinate handling. Kornia also supports augmentations in the GPU that takes advantage of parallelization. A noteworthy feature of the Kornia data augmentation module is that it allows the user to retrieve the applied transformations after each call. This is particularly helpful when randomized transformations are applied and the transformation must be tracked during the training process. Finally, Kornia supports auto-augmentation policies [34] where data augmentation transformations have been previously learned on existing datasets and can be applied to new, similar data. Kornia augmentations are divided into four main groups: Transforms2D, Transforms3D, Normalization, and Resize. The transforms groups are further divided into Intensity, Geometric, and Mix. Considering all the categories, the total number of aumentation techniques is 64.

### 2.10. SOLT

SOLT [30] is an highly parameterized data augmentation library with OpenCV [32] in the backend. It supports images, segmentation masks, labels, and key points. It is compatible with PyTorch and boasts clear and comprehensive documentation as well as ease of extension. SOLT uses a DataContainer to wrap all your data into a single object. This acts similarly to imgaug, however, with faster run times. This enables the application of the same transformation to multiple images (e.g., to a minibatch of images, an instance segmentation mask, a set of keypoints). All that is needed is the data and whether it is an image (I), mask (M), key points (P), or labels (L). Considering only the augmentation approaches for images, the number of supported techniques is 21.

### 2.11. Torchvision

Torchvision [31] is a Python library used in conjunction with the PyTorch machine learning framework. The library offers several functionalities as python modules. Specifically: torchvision.datasets allows to access different datasets to be used for training; torchvision.io supports a wide range of backends to deal with image and video input/output; torchvision.models gives access to models and pre-trained weights for different computer vision tasks; torchvision.ops implements efficient operators specific for computer vision problems; torchvision.utils contains miscellaneous functionalities mostly for data visualization; and torchvision.transforms allow the user to perform data transformations on images, bounding boxes, masks, and videos for data augmentation. The module is flexible and the supported transformations can be easily chained together to compose a composite transformation pipeline. Torchvision also support auto-augmentation policies [34]. Since version 0.8, Torchvision supports data transformation on the GPU. The number of augmentation techniques included in the Torchvision library is 27.

## 3. Data Augmentation Taxonomy

Data augmentation techniques can be divided according to the task, the problem, or the way that they operate on the images. Alomar et al. [4] propose a taxonomy of DA techniques, divided into Traditional and Deep Learning techniques. Traditional techniques are further subdivided into Geometric, Photometric, Noise, and Kernel techniques. In the class of Deep Learning techniques, are Generative Adversarial Network (GAN) and Style Transfer. This taxonomy is not exhaustive since it does not include a large part of the techniques that are considered in this work. Kebaili et al. [17] provide more insight into deep learning techniques; their work examines the application of deep generative models for data augmentation in medical image analysis. Specifically, three types of deep generative models for medical imaging augmentation are examined: variational autoencoders, adversarial generative networks, and diffusion models.

**Figure 2:**
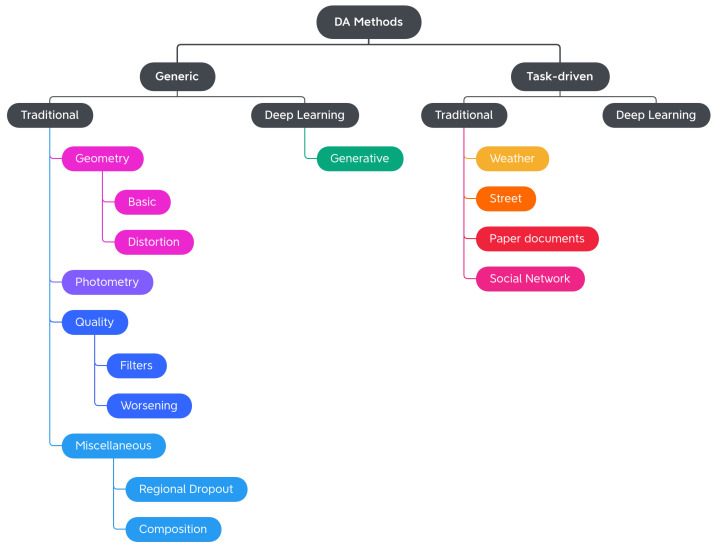
High-level overview of the proposed Computer Vision Data Augmentation taxonomy.

In this work, we propose a more exhaustive taxonomy (Figure 2) of data augmentation techniques, based on the methods available in the major public data augmentation libraries for image processing. Edge detection techniques, which Alomar et al. grouped in the Kernel class, have not been considered in this work. Their use, in fact, appears to be more appropriate for preprocessing operations than data augmentation, since they distort the images in terms of internal shapes and color. For the same reason, the methods of converting between color spaces and thresholding operations were also excluded from the analysis.

The analysis of the 11 libraries yielded a long list of traditional image augmentation techniques. Using the classifications mentioned above as a starting model, we decided to categorize the traditional techniques on the basis of the type of effects obtained on the augmented image. Various types of techniques have been encountered: basic geometrics, geometric distortion, photometrics, blur filters, sharpness filters, mathematical morphology, style filters, noise injection techniques, worsening techniques, tessellation techniques, regional dropout, composition techniques or mixed samples, and techniques that augmented images specifically for certain purposes. We decided to carry out a first subdivision of the techniques, differentiating generic transformations from those dedicated to specific tasks in specific domains, such as adding a manhole cover to a street image or adding a template that mimics a bookbinding to a scanned document image. We have, therefore, created the two classes *Generic* and *Task-driven*. Both classes are subdivided into *Traditional* and *Deep Learning*.

Traditional Generic techniques are divided into: (i) *Geometry*, which contains techniques that change the position, orientation, or shape of objects; (ii) *Photometry*, which includes photometric techniques, which modify the photometric properties of the image, (i.e., brightness, contrast, and color); (iii) *Quality*, in which we have included blur filters, sharpening filters, mathematical morphology operations, style filters, noise injection techniques, generic worsening techniques, and tessellation methods (i.e., all those changes that modify the image in terms of quality); (iv) *Miscellaneous*, which contains the regional dropout, composition, and mixed sample techniques.

The internal classification of these classes was then perfected, determining the various subcategories: Geometry has been divided into *Basic Geometry*, which contains the simplest and best known geometric transformations, and *Distortion*, which contains techniques that deform the image by modifying the internal geometry; Photometry has no subcategories, but we wanted to highlight that although many libraries include methods that allow you to modify the contrast in a generic and immediate way, there are also more specific techniques to achieve the same goal; Quality is divided into *Filters* and *Worsening*. Filters contains blur filters, sharpening filters, mathematical morphology techniques, and what we have defined as style filters. Worsening contains techniques that aim exclusively at image deterioration and contains the subcategories of noise injection and tessellation; Miscellaneous is divided into *Regional Dropout* and *Composition*. In the latter, there is a further subcategory that includes the various types of Mixed Sample techniques.

The task-driven techniques encountered in the analysis of the libraries are traditional ones. They have been divided according to the fields of use: *Weather* contains techniques involving image effects that simulate atmospheric phenomena such as rain or fog; *Street* includes methods that add street elements such as gravel or manhole covers to the image; *Paperdocs* encompasses paper document methods, which produce realistic and dirty copies of paper documents from loose copies of artifacts; *Social Network* includes transformations related to social networks, which produce images similar to those typical of social networks, such as captured screenshots or memes.

No deep learning techniques were encountered during the analysis of the libraries. The inclusion of this group in the taxonomy and the discussion in Section 4.3 are given for completeness.

Table 3 lists the different augmentation techniques included in the eleven libraries according to the proposed taxonomy. In brackets, are some references to the underlying rationale of the approaches. If the reader were to consult the documentation of any of the analyzed libraries, they would probably notice that the given classes are much more numerous than the listed techniques. The implementation of a data augmentation technique, in fact, can take on multiple forms, giving rise to a wide range of possible methods. In order to make the consultation more user-friendly, the column of labels does not contain the list of all classes, but rather the transformations (or the group of transformations) associated with them. For a more detailed view, please refer to the corresponding table available at DALib website: http://www.ivl.disco.unimib.it/activities/dalib/ (accessed on 10 October 2023).

Taxonomy details will be explored in the next section, where the techniques will be further described.

## 4. Data Augmentation Techniques

In this section, we describe in more detail the data augmentation methods available in the considered libraries, organized according to the taxonomy previously defined in Section 3 and graphically depicted in Figure 2. The methods reported here are the ones available at the date of publishing, for an up-to-date list, please see the DALib website.

### 4.1. Generic Traditional Techniques

#### 4.1.1. Geometry

**Figure 3:**
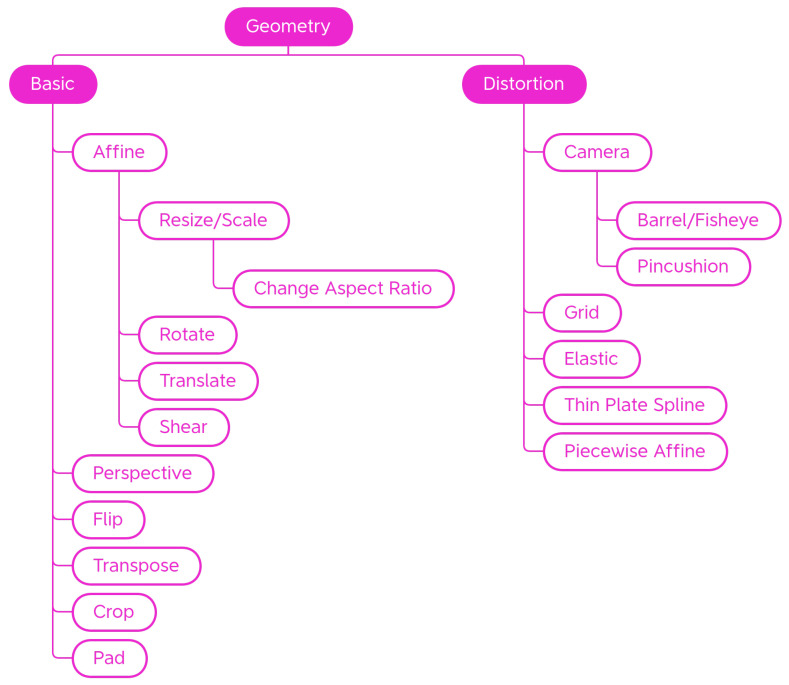
Taxonomy of the Geometry—generic traditional data augmentation techniques class.

Inside the Geometry class, are *Basic* transformations that alter the shape, position, or orientation of image elements as well as *Distortion* geometric transformations. Transformations are organized in subcategories as shown in Figure 3.

##### Basic

**Figure 4:**
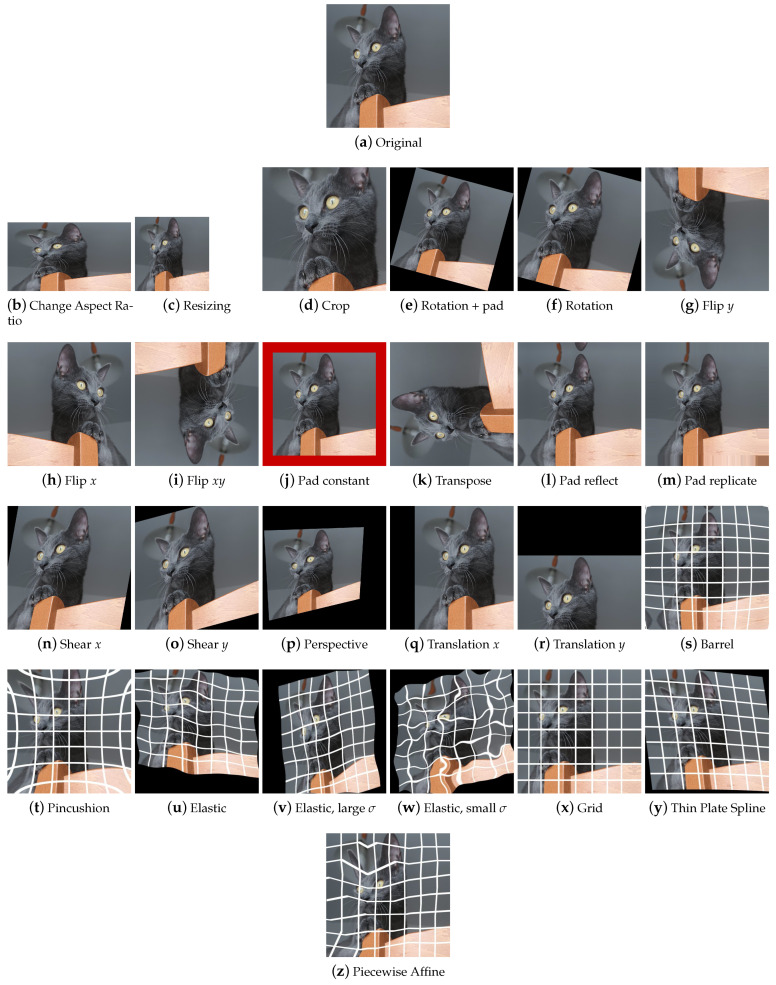
Effects of the various Geometry transforms on a sample input image. Grid superimposed for readability.

Basic geometric transformations are an effective and commonly used set of methods for providing large numbers of augmented data samples. Figure 4 shows some examples of the effect of those transforms on a sample input picture.

**Resizing** To easily increase the variations in the dataset, the images can be magnified or shrunk (Figure 4b,c); if the aspect ratio is not kept constant, the image may be slightly or visibly distorted; the upscaling and subsequent downscaling of the image is used as a worsening transform.

**Rotation** Rotation (Figure 4e,f) is used to change the orientation of image elements. The rotation functions that are implemented in the libraries can allow the input of parameters of choice, be random, or have fixed degrees of rotation generally set at 90°, 180°, and 270°. Unless combined with a crop, the rotation transform produces areas of black pixels. To preserve all the parts of the original image, some libraries provide the possibility to produce the output image with dimensions bigger than the starting dimensions and this also happens for other techniques, such as shear.

**Translation** A translation (Figure 4p,q) can be used to change the position of an image element. It has the side effect of losing part of the image. Translation can be applied on a single image axis or on both axes.

**Shear** Shear (Figure 4m,n) and perspective projections are—after aspect ratio change—the simplest distortion transformations of an image. Shear mapping shifts each point in the image along the chosen *x* or *y* axis, proportional to their position relative to the other axis. In some libraries, the more generic term *Skew* is present, which sometimes indicates the shear transformation—as in Augly—while at other times, it indicates the perspective transformation—as happens in Augmentor.

**Perspective** When an object from the real world is captured, it can appear differently depending on the camera point of view. Perspective transformations (Figure 4o) manipulate the image to simulate different viewing angles. The result is an image that appears to have been taken from a different viewpoint. As mentioned above, this transformation is present in the Augmentor library under the name of Skew.

**Flip** The flip transformation (Figure 4g–i) flips the image around a reference axis. It is a simple but powerful technique that, however, cannot be used in contexts where asymmetries play an important role, such as in text recognition.

**Transpose** Transpose swaps rows and columns. It is a quick way to achieve the result that would be obtained by performing a horizontal flip and a subsequent rotation of 90 degrees counterclockwise.

**Crop** With the cropping technique (Figure 4d), a subset of the original image is obtained. This can help a machine learning model by isolating an object of interest by removing the uninteresting parts of the image. However, it is wise not to abuse this transformation because there is the risk of eliminating meaningful parts of the image.

**Pad** The pad transform (Figure 4j–l) extends the image by adding pixels along the edges. The added pixels can be of a fixed color or be obtained by reflection or replication from the original image. This technique is often used to integrate the rotation, translation, shear and perspective projection methods, i.e., those transformations that have the production of black pixels as a side effect: to limit the number two non-informative pixels, some padding can be applied to complete the image.

##### Distortion

Distortions are mostly used in the field of handwriting recognition [38] or medical imaging [65,66]. In addition to scaling, shearing, and perspective transformation, there are a number of distortions that modify images in different ways. Figure 4 shows some examples of the effect of those transforms on a sample input picture. For these transformations, a regular grid is superimposed for better readability of the final effect.

**Camera distortions** This groups distortions commonly introduced by camera lenses. Barrel (Figure 4s) distortion causes straight lines to appear curved and pointing outward from the center of the image; since this type of distortion is obtained in photography with wide-angle lenses, we can find it in some libraries under the name of Fisheye. Conversely, the Pincushion distortion (Figure 4t), the distortion that can be obtained in photography with the use of telephoto lenses, causes the lines to curve towards the inside of the image.

**Elastic Transform** The Elastic Transform [38] (Figure 4u) operates as follows: the random displacement fields Δx(x,y) and Δy(x,y) are generated, i.e., the sets of displacements of the image points on the x and y axes, and are convoluted with a Gaussian of standard deviation σ, where σ equals the elasticity coefficient. With a large σ the resulting values will be very small (Figure 4v); if we normalize, the displacement field will be close to constant, with a random direction. Conversely, with σ small, the field will appear as a completely random field after normalization (Figure 4w). With intermediate values of σ, displacement fields appearing as elastic deformations. The displacement fields are then multiplied by a scale factor α which controls the amount of deformation.

**Grid distortion** Grid distortion (Figure 4x) is a technique in which the image is mapped to a grid structure whose cells are resized. This leads to a peculiar deformation of the image.

**Thin Plate Spline** Let us imagine we have a thin metal plate on which we can place some points, which we define as control points. If the plate is moved or deformed in a certain way, the control points will follow the movement. The Thin Plate Spline (TPS) technique (Figure 4y) relies on this physical analogy to calculate plate deformation and determine how control points move. To warp an image, two sets of control points are selected, one in the source image and one in the destination image. The idea is to find a transform that matches the control points of the original image to those of the destination image. This transformation will then be applied to all pixels of the image to obtain the desired deformation.

**Piecewise Affine** The Piecewise Affine transformation (Figure 4z) places a regular grid of points on an image and randomly moves around these points via affine transformations. This leads to local distortions in the image.

#### 4.1.2. Photometry

**Figure 5:**
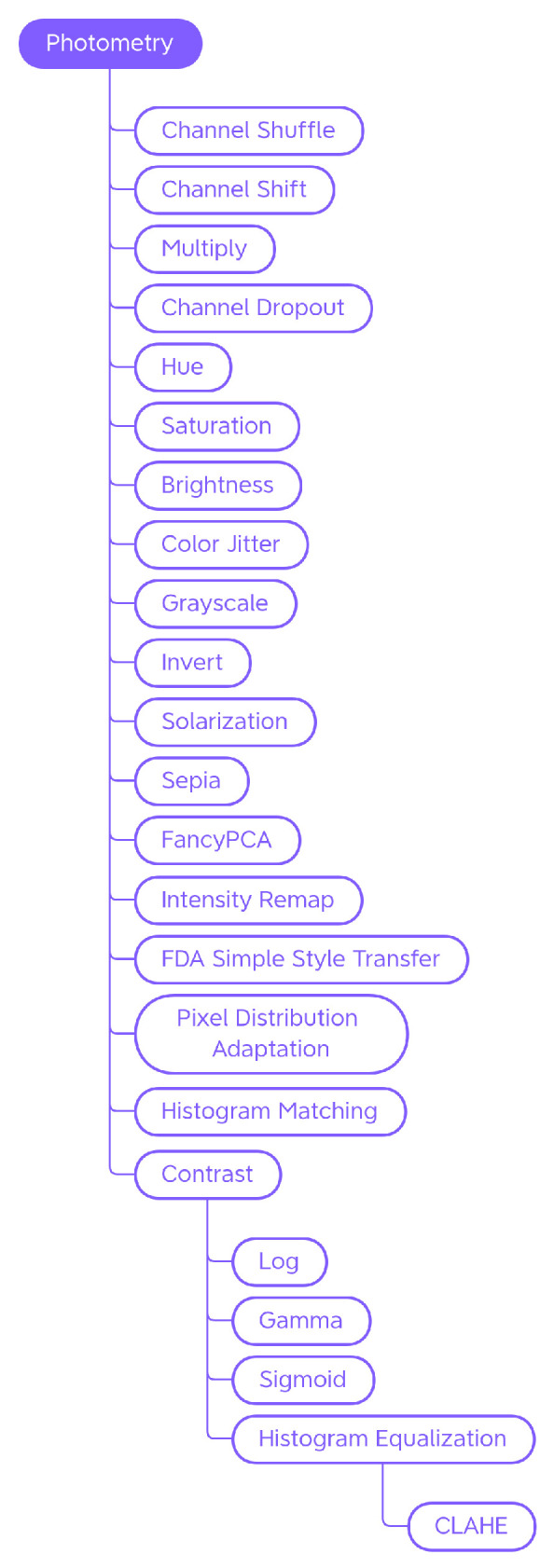
Taxonomy of the Photometry—generic traditional data augmentation techniques class.

**Figure 6:**
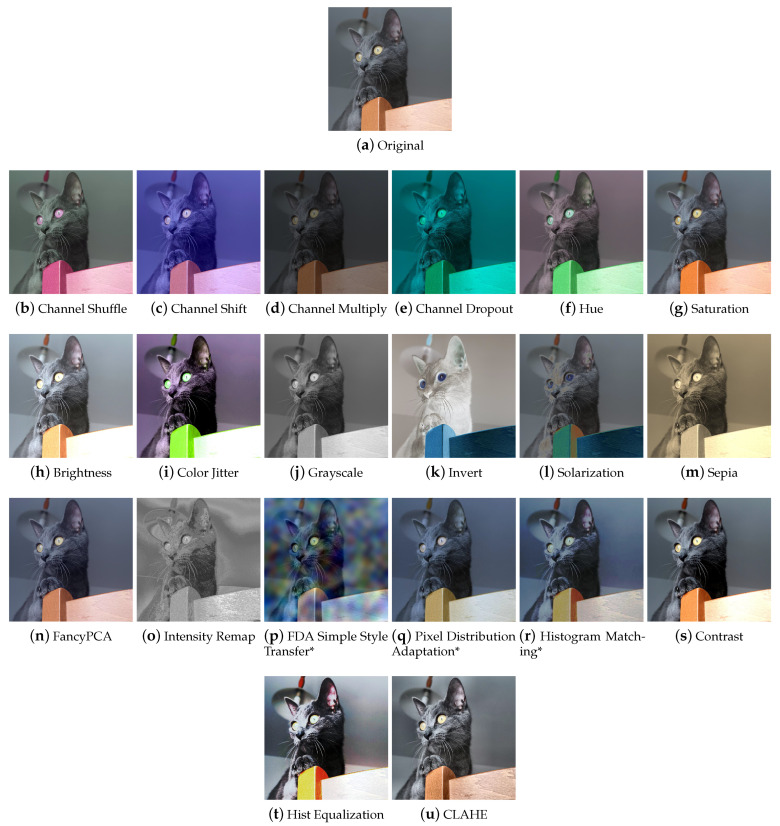
Effects of the major Photometry transforms on a sample input image. * Van Gogh’s Starry Night used as target image.

Photometric techniques modify images in terms of color, brightness, and contrast by controlling the pixel values in relation to a specific color space (Figure 5). Figure 6 shows some examples of the effect of those transforms on a sample input picture.

**Channel Shuffle, Shift, Multiply, and Dropout** Within a chosen color space, it is possible to randomly rearrange the order of the channels (Channel Shuffle (Figure 6b)), modify the pixel values by multiplying them by a factor (Multiply (Figure 6c)), shift the channel values by a certain amount (Channel Shift (Figure 6d)), or eliminate—with consequent loss of information—one or more channels (Channel Dropout (Figure 6e)).

**Hue, Saturation, Brightness, and Color Jitter** By manipulating the HSV color space channels (Figure 6f–h), it is possible to change the hue, saturation or lightness values of the image; the augmentation technique that randomly modifies these values is called Color Jitter (Figure 6i).

**Grayscale** By bringing the saturation values to zero, it is possible to obtain a grayscale image, however the Grayscale transformation (Figure 6j) can be implemented in other ways, for example, by calculating the mean of the RGB channels and replacing them with a single channel. Actually, since the colors red, green, and blue have different perceived brightness, a weighted average of the channels is calculated where green weighs about 59% and red 30%. Therefore, it is possible for Grayscale methods of different libraries to give different results.

**Invert** The Invert technique (Figure 6k), also known as Negative, inverts the values of each channel of the image.

**Solarization** Solarization (Figure 6l) is a photographic phenomenon that occurs when photographic film is excessively overexposed, causing an inversion of tones in the resulting image. The Solarization transform mimics this phenomenon by setting a threshold value and inverting values below or above it.

**Sepia** The Sepia augmenter (Figure 6m) modifies the image to give it a warm, retro look, mimicking photos that have been sepia-toned.

**FancyPCA** FancyPCA (Figure 6n) alters the intensity of the RGB channels by performing Principal Component Analysis on the color channels, as described in [47]. This technique allows images to be augmented so that they retain the main color characteristics but with a controlled variation.

**Intensity Remap** Intensity Remapping replaces the pixel values of the original image with values obtained via an intensity remapping curve (Figure 6o). The exact formulation of the operation can be found in [45].

**FDA Simple Style Transfer** Style Transfer, as we will discuss later, is a technique by which it is possible to manipulate the visual aspect of an image in order to transfer the style of another image to it (Figure 6p). Fourier Domain Adaptation is a technique that uses a Fourier transform to adapt images from one domain to another. The Albumentations FDA technique allows the user to perform a sort of Style Transfer through the Fourier Transform. FDA needs no deep networks for style transfer, and involves no adversarial training. For further information on this technique, please read [67].

**PixelDistributionAdaptation** Albumentations’ PixelDistributionAdaptation is a technique that uses simple transformation methods such as PCA (Principal Component Analysis), StandardScaler, or MinMaxScaler to scale the pixel distribution of the original image to match the pixel distribution of a reference image (Figure 6q).

**Histogram Matching** Histogram Matching is a technique that manipulates the pixels of an input image so that its histogram matches the histogram of a reference image. It is a simple example of style transfer that changes the color mood of an image (Figure 6r).

##### Contrast

The contrast (Figure 6s) can be calibrated using the Log, Gamma, and Sigmoid functions, or by adjusting the histogram of the color channels (equalizing it or making it correspond to a reference histogram).

**Logarithmic scaling** Logarithmic scaling expands the range of dark values and compresses the light ones and has the formula s=clog(1+r), where *c* is a constant, *s* and *r* are the pixel values, respectively, outgoing and incoming, and it is assumed that r≥0.

**Gamma Correction** The Gamma Correction has the formula $s=cr^{\gamma }$. For values of γ<1, it expands the blacks and compresses the whites, while for values of γ>1, it expands the whites and compresses the blacks.

**Sigmod function** The Sigmod function is used to adjust contrast non-linearly, resulting in an “S” shaped curve. The formula is $s=1/(1+e^{-k(r-c)})$, where *k* is a slope factor and *c* is a translation point. By increasing or decreasing the value of *k*, the slope of the curve and, therefore, the contrast can be adjusted. By modifying the value of *c*, the curve can be translated along the *x* axis, adjusting the brightness of the image.

**Histogram Equalization** Histogram Equalization (Figure 6t) is a technique used to increase the contrast of an image. If the histogram of an image uses only a small part of the range of gray values, a low-contrast image results. Through equalization, the histogram will use the entire intensity range and the image will be have more contrast.

**CLAHE** Traditional equalization affects the entire image and this can lead to noise amplification. Contrast Limited Adaptive Histogram Equalization (CLAHE) (Figure 6u) addresses this issue; it divides the image into small regions, called blocks, and applies histogram equalization to each block separately. This type of processing preserves the details and local characteristics of the image. CLAHE also introduces a contrast limiter, which prevents over-amplification of already high-contrast images. This helps produce more natural-looking images.

#### 4.1.3. Quality

**Figure 7:**
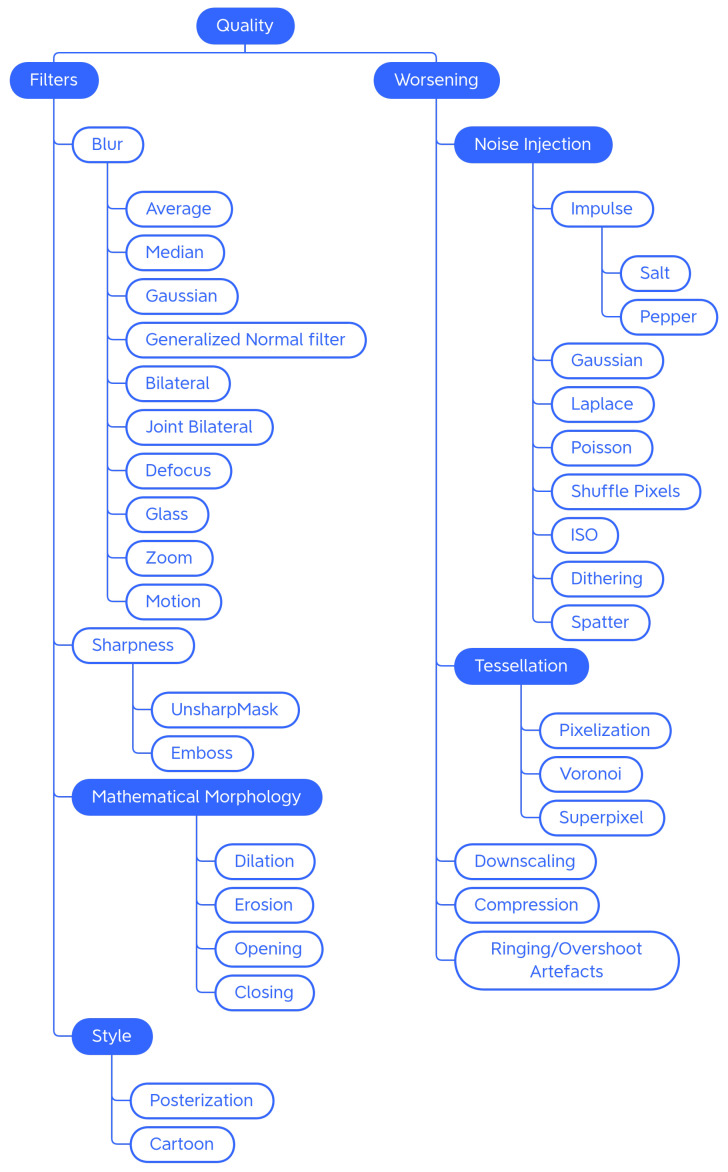
Taxonomy of the Quality—generic traditional data augmentation techniques class.

In the Quality category, we placed the methods that are used to improve or worsen the images (Figure 7). In the context of DA, the most-used techniques are the ones that make the image worse (e.g., to train Super Resolution models [50]).

##### Blur Filters

Blur or Smooth filters are used in image processing to blur an image or a portion of it. They can be applied to enhance the image by reducing noise, to create artistic effects, or to degrade the image quality.

**Figure 8:**
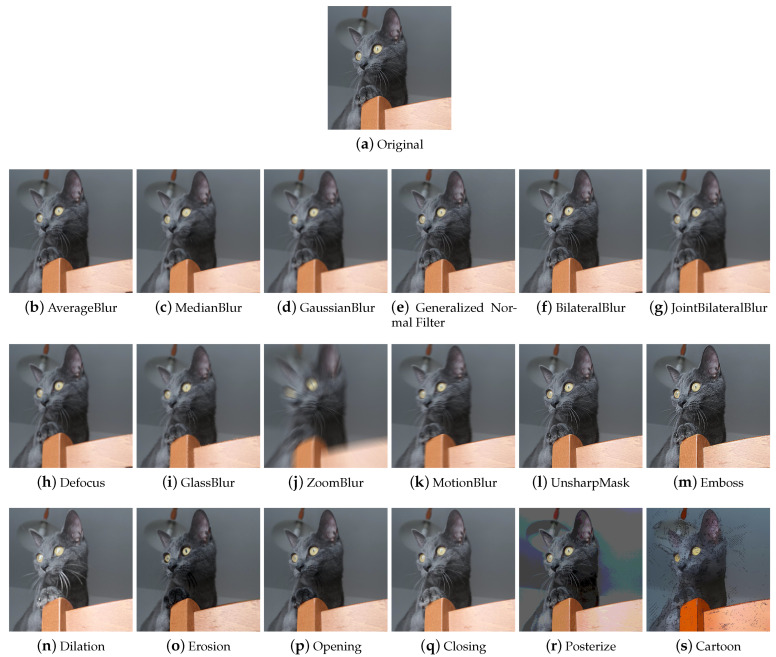
Effects of some of the Quality transformations on a sample input image.

**Average** Average blurring (Figure 8b) is an operation that, having defined the size of the kernel, replaces each pixel with the value obtained by calculating the arithmetic mean of the values of the pixels included in its neighbourhood; the new pixel values may not be present in the original image.

**Median** The Median filter (Figure 8c) is typically used to remove impulsive noise in image enhancement tasks. It replaces each pixel with the median value of the pixels in the neighborhood considered. The new pixel values are therefore also present in the original image; no new intensity values are produced.

**Gaussian** The Gaussian filter (Figure 8d) has the particularity of having the weights distributed following a Gaussian distribution in that the central value has the highest value and as one moves away from the center, the values decrease with a speed that depends on sigma—the larger the sigma, the slower the decay rate. The filter size and sigma are related to each other. Unlike an average filter, the Gaussian filter places more weight on the values of the pixels near the center of the filter. This results in a filter that, with the same dimensions, applies a blur less strong than an average filter. The possibility of varying the sigma also allows greater control over the final result. Very low sigmas give a very concentrated filter (at the very least, you have an identity filter). Very high sigmas give a flat filter (at the average limit).

**Generalized Normal filters** Generalized Gaussian filters (Figure 8e) are based on the generalized Gaussian probability distribution. The generalized Gaussian distribution is a generalization of the Gaussian distribution and differs from the latter in having a wider or narrower shape and can be asymmetric.

**Bilateral** The Bilateral filter (Figure 8f) reduces image noise while preserving the edges. It calculates a weighted average of the pixel values in the image, assigning different weights based on the spatial distance and the difference in intensity of the pixels. Spatial distance measures how close the pixels in the image are to each other, while similarity of pixel values indicates how similar the pixel values are to each other. Pixels that are closest to a given pixel will have more weight on average, while pixels with significant differences in intensity will have less or no weight.

**Joint Bilateral** The Joint Bilateral filter (Figure 8g) extends the concept of the bilateral filter. The only difference is that the Gaussian kernel of the color is calculated based on another image called the guide image.

**Defocus** The Defocus technique (Figure 8h) simulates the look of an image that is out of focus.

**Glass** Glass blur (Figure 8i) produces an image that appears to be seen through frosted glass. To achieve this particular effect, an initial version of the image is created by applying a Gaussian blur filter. Subsequently, pixel displacements are applied to the image based on the selected mode (“fast” or “exact”). These displacements create a distortion effect within the image. After the number of iterations chosen by the user, another Gaussian blur filter is applied to the resulting image to make the blurring effect more uniform and smooth.

**Zoom** The zoom burst is a photographic technique that can be obtained with lenses equipped with manual adjustment of the focal excursion for which the image has a typical radial blur, in which it seems that the center (single focus point) approaches the observer at high speed. The Zoom Blur (Figure 8j) mimics this photographic effect.

**Motion** This technique (Figure 8k) replicates the effect that occurs when a moving object is photographed that moves very fast or is shot with long exposure times.

##### Sharpening Techniques

Sharpening techniques are employed to improve edge definition and detail, making the overall image sharper and more defined. They tend to be used to improve the visual appearance of blurry or poorly detailed images. Generally, the libraries include methods generically described as capable of increasing the sharpness of the image, without the procedure being explicitly explained. It is, therefore, possible that sharpening kernels are used, such as the Laplace filter.

Further sharpening techniques available within the libraries are Unsharp Masking and the Embossing technique.

**Unsharp Mask** Unsharp masking (Figure 8l) is a technique used to sharpen the image and emphasize details. It works as follows: a blurring filter is applied to the starting image; subsequently, the blurred image is subtracted from the original image in order to isolate the details; finally, the resulting image of the details is added to the original image for greater sharpness. This technique tends to introduce overshoot artifacts.

**Emboss** Embossing (Figure 8m) is a technique that produces an embossed effect on the image through the use of embossing kernels. The image augmentation Emboss technique produces an image with emphasized and raised details, obtained by adding the original image to the image obtained through embossing.

##### Mathematical Morphology

Mathematical morphology (MM) is an image processing technique that relies on morphological operators to modify the shape, structure, and characteristics of an image. It is typically used on binary images to separate objects, remove noise, or fill holes. Math morphology can be applied on RGB images by converting them to grayscale first. In grayscale MM, an image is treated as a three-dimensional surface, with the pixel intensity representing the third dimension. The main focus is the analysis and manipulation of the shape and structure of the object represented by the image. The main operations of mathematical morphology are described below.

**Dilatation** Grayscale dilation (Figure 8n) replaces each pixel with the maximum value among the pixels in its neighborhood, which has the effect of expanding the lightest regions of the image. It can be used to fill gaps or join regions of similar intensity.

**Erosion** Grayscale erosion (Figure 8o) consists of replacing each pixel in the image with the least value among the pixels in its neighborhood. This has the effect of smoothing out the lightest regions of the image. This technique can be useful for reducing noise in images, as it tends to eliminate regions of lower intensity.

**Opening** Opening (Figure 8p) an image in grayscale involves performing a grayscale erosion followed by a grayscale dilation.

**Closing** Closing (Figure 8q) an image to grayscale involves performing a grayscale dilation followed by a grayscale erosion. By appropriately choosing the dimension of the structuring element based on the dimensions of the image, the techniques of mathematical morphology produce peculiar effects on the image that decrease the quality of the image and, in a certain way, modify its overall brightness values; therefore, applying grayscale mathematical morphology to a color image requires consideration of the color implications.

Another effect that catches the eye is the corruption of the internal shapes: the augmented image seems to be composed of many small messy patches, of shape and size deriving from the structuring element. Overall, the image is degraded and contains much less detail than the original image.

##### Style Filters

Within this category, have been placed the techniques that transform the appearance of photos or images to obtain artistic, stylized, or caricatured results.

**Posterization** The posterization (Figure 8r) technique is a compression and image deterioration technique, which in some contexts, can be used for the artistic effect it produces on the images. It reduces the number of tonal gradations present in the image, resulting in an image that has a cartoon-like appearance.

**Cartoon** The Cartoon augmenter (Figure 8s) converts the image style to a more cartoonish one. This technique is only available in the imgaug library. To obtain this particular effect, the image is first blurred with a median filter; subsequently, it is divided into homogeneous regions using a mean-shift based segmentation algorithm. An edge detection method is then applied to identify edges in the image; sometimes, there may be areas with too many edges detected; these unwanted edge regions are suppressed. The saturation of the image is increased and finally, the detected edges are combined with the original image, completing the cartoon-style transformation.

##### Noise Injection

Adding noise to the images is done with the aim of making the model more robust and less sensitive to small variations or perturbations in the input data. This makes the model better able to generalize correctly on new data that might have similar noise or variation.

**Figure 9:**
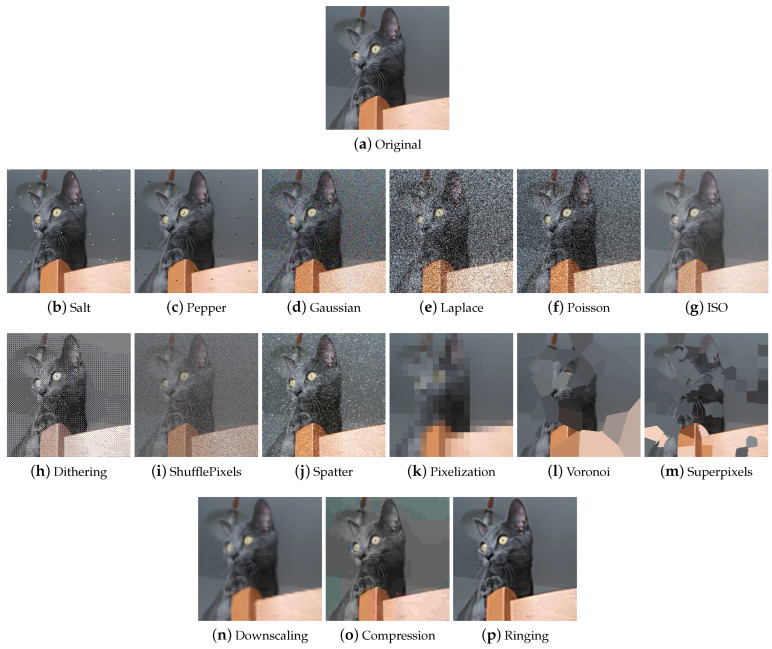
Other effects of some of the Quality transformations on a sample input image.

**Impulse, Salt and Pepper** Impulse noise is the presence of white (Salt) (Figure 9b) and black (Pepper) (Figure 9c) pixels in the image and is usually present in images due to noise in signal transmission or sampling errors. Other names by which this type of noise is known are bipolar impulse noise (unipolar if either Salt or Pepper), data-drop-out noise, and spike noise.

**Gaussian** Gaussian noise (Figure 9d) is characterized by having a probability distribution that follows the bell-shaped curve of the normal distribution. The presence of this noise in digital images is typically due to camera sensor noise which can be caused by low light or high temperature, or by electronic circuit noise.

**Laplace** Laplace noise (Figure 9e) follows a Laplace distribution. Furthermore, called the double exponential distribution, this distribution has the shape of a kind of pointed bell.

**Poisson** Poisson noise (Figure 9f) follows a Poisson distribution.

**ISO** ISO noise in digital images (Figure 9g) refers to unwanted distortion or interference that appears as dots or grain in the image and is due to the high ISO sensitivity used in the camera. The technique of the same name modifies the image by adding noise so that it appears that the original photo was already corrupted with ISO noise.

**Dithering** Dithering (Figure 9h) is a technique that is used to reduce the banding effect that can occur when reducing the depth of colors. Banding refers to the appearance of visible streaks or gradations between colors, especially in areas of tonal transition. Dithering works by deliberately adding random noise to the image; this noise makes it more difficult for the human eye to detect sharp color transitions, creating a smoother gradient effect.

**Shuffle Pixels** The Shuffle Pixels technique (Figure 9i) injects noise into the image by randomly swapping pixels.

**Spatter** The Spatter augmenter (Figure 9j) deteriorates the image by simulating the occlusion of a lens that occurs when the lens is dirty with mud or rain.

##### Tessellation

Tessellation is used as a data augmentation technique for degrading images. It is advisable to pay particular attention when entering any parameters to the choice of the size of regions: regions that are too large could lead to large losses of information. The tessellation techniques encountered during the analysis of the libraries are described below.

**Pixelization** Pixelization (Figure 9k) is a technique by which the resolution of an image is reduced by replacing groups of neighboring pixels with a single pixel or, to maintain the same dimensions, with a section of pixels of constant color. The color of the pixel generally coincides with the average of the colors of the neighborhood that will be replaced.

**Voronoi** Voronoi tessellation is a special type of subdivision of a space into polygons, done so that each cell contains a specific data point and all points in the same polygon are closer to that data point than any other point in the diagram. Voronoi (Figure 9l) tessellation can be used to achieve the same effect as pixelization or a similar effect but with rectangular rather than square cells.

**Superpixel** The Superpixel technique (Figure 9m) is used to group similar pixels into compact, homogeneous regions. There are several superpixel segmentation algorithms including SLIC (Simple Linear Iterative Clustering), Felzenszwalb, and Watershed. Each algorithm has its own parameters to adjust based on the specific needs of the application.

##### Other Worsening Techniques

In addition to noise injection and tessellation techniques, there are further augments to degrade images, which will be described below.

**Downscaling** The term downscaling generally refers to the process of reducing the size or resolution of the image (Figure 9n). The Albumentations library contains the Downscale technique, which involves scaling down (downscaling) followed by restoring the original size (upscaling). Downscaling results in the loss of detail and information due to the reduction in resolution. Although the image is restored to its original size, since the loss of information during downscaling cannot be completely restored, the resulting image may show artifacts, blurring, or lower overall quality than the original image.

**Compression** Compression (Figure 9o) is a technique used to reduce the file size of digital images. Various algorithms for image compressions exist; common ones adopt a lossy compression strategy, which drastically reduces the amount of data used to encode the image at the cost of lower image quality.

JPEG compression detects and exploits the visual characteristics of the image, such as spatial redundancy and human perception of color, to eliminate or reduce non-essential information. JPEG compression is divided into two stages, encoding and decoding. During encoding, the image is decomposed into 8×8 pixel blocks; a transformation called Discrete Cosine Transform (DCT) is applied to each block, which converts the image from the spatial domain to the frequency domain, so that frequency information can be represented more efficiently. Next, quantization techniques are applied, which reduce the accuracy of the frequency components of the image. This helps reduce the amount of data needed to represent the image. During the decoding stage, the image is reconstructed using frequency information and quantization values. The amount of JPEG compression can be controlled by selecting a desired quality level during the compression process. A higher quality level will result in less compression and greater visual fidelity to the original image, but will also result in a larger file size. Conversely, a lower quality level will result in higher compression and smaller file size, but also higher quality loss.

Another type of compression encountered when parsing libraries is WebP compression. This uses several strategies to reduce file size: lossy compression, lossless compression, or prediction-based compression, where image information is analyzed and correlations between surrounding pixels are used to further reduce file size. WebP allows you to reduce the size of image files without a significant loss in visual quality.

**Ringing/Overshoot Artefacts** Ringing artifacts (Figure 9p) appear as a series of bright and dark lines or rings around a high-contrast object, such as a sharp border between differently colored objects.

Overshoot artifacts are characterized by peaks or patterns that appear around high-contrast edges or transitions.

#### 4.1.4. Miscellaneous

**Figure 10:**
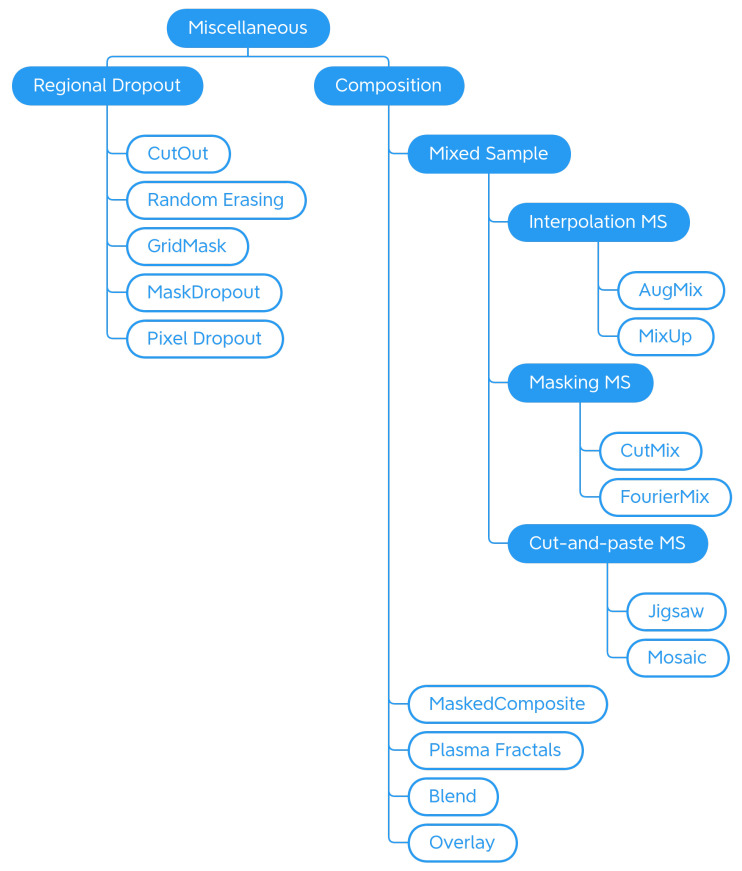
Taxonomy of the Miscellaneous—generic traditional data augmentation techniques class.

Is the last category of traditional generic data augmentation techniques and groups, various effects that do not fit any other previously introduced section (see Figure 10).

##### Regional Dropout

**Figure 11:**
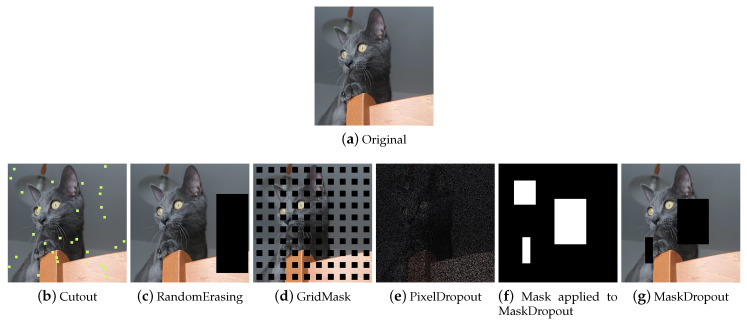
Example of how Regional Dropout techniques work on a sample image.

Regional Dropout (Figure 11) refers to a set of techniques that remove information pixels by overlaying on the training images patches of generally black or noisy pixels. Information dropping techniques improve generalization and localization by letting a model attend not to only the most discriminating parts of objects but rather to the entire object region [58]. Although these techniques help to make the models more robust, it is wise to pay attention to the size of the patches, as the larger the patch, the greater the risk of incurring the loss of important information and ineffective training.

**Cutout** Cutout [53] (Figure 11b) selects a random rectangular region of the image and replaces the pixels with black pixels or a patch of noise. The technique is implemented differently in the various libraries, allowing not only the user to choose the patch filling mode but also a filling color, the number of patches, and their size. Although it originated as a technique to allow the model not to focus on specific features, in some cases, when patches are small and numerous, the implementation is similar to impulsive noise and this makes these derived augmentations also useful for gaining robustness against image corruption.

**Random Erasing** Random Erasing [51] (Figure 11c) also selects a rectangular region of the image and replaces its pixels. It provides for a single region to be deleted, the size of which can vary. Pixels are deleted or replaced with noisy patches.

**GridMask** GridMask [52] (Figure 11d) is a variant of Cutout that applies a grid-shaped mask to the image. This mask can be customized in terms of the size of the dropped squares and their distance from each other. Some libraries implement the technique allowing the user to rotate the mask or invert it.

**MaskDropout** Taking an image and a mask as input, MaskDropout (Figure 11g) zeros out image regions corresponding to randomly chosen object instances from the mask.

**PixelDropout** PixelDropout (Figure 11e) randomly chooses some pixels of the image and deletes them, setting their value to zero or to a specific value entered by the user.

##### Composition

**Figure 12:**
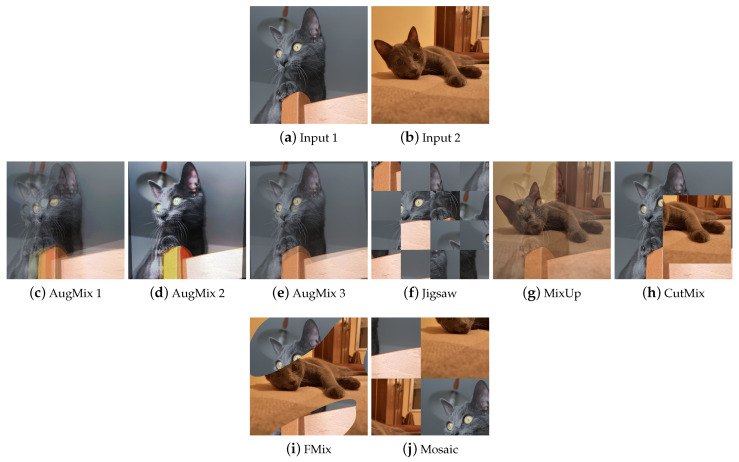
Examples of Mixed Sample Data Augmentation. (**a**,**b**) are the input images. The Input 1 image is used for AugMix (**c**–**e**), and Jigsaw (**f**). The remaining augmentations (**g**–**j**) use both input images mixed in different ways.

The Composition class includes techniques that produce an image from the combination of multiple elements, such as an image with a mask or an image with another image or an image with a copy of itself, in a different way from the techniques seen previously. This class includes Mixed Sample (MS) data augmentation techniques (Figure 12), which consist of synthesizing an image starting from multiple (usually two) samples by combining both sample values and labels in a linear combination (Interpolation MS) or by the use of a mask (Masking MS), or with a cut and paste (Cut-and-paste MS).

**AugMix** AugMix [55] (Figure 12c–e) takes a single image as input. From a predefined set of augmentations, transformations are stochastically chosen and applied to the input image to generate augmented images. These are then mixed together to produce the final augmented image.

**Jigsaw** Jigsaw (Figure 12f) divides the input image into cells of the same size; a random permutation of cells is generated, and the image is reconstructed by shuffling the cells in the order defined by the permutation. The number of cells is user-definable. In imgaug, the maximum number of steps for each cell can also be defined.

**MixUp** MixUp [56] (Figure 12g) takes two images as input and interpolates them by performing the weighted sum. It differs from methods like Kornia’s *AddWeighted* in that it also mixes labels.

**CutMix** CutMix [58] (Figure 12h) was created to overcome the problems of the regional dropout and Interpolation MS methods; i.e., the presence of uninformative pixels and the unnaturalness of the samples, respectively. It creates a rectangular-shaped binary mask and combines two input samples according to this mask.

**FMix** FMix [59] (Figure 12i) maximizes the number of possible masks by sampling a low-frequency grayscale mask from Fourier space, which is converted to binary with a threshold. The resulting mask is then used to combine the two input images together.

**Mosaic** Mosaic (Figure 12j) is implemented by the Kornia library as follows: the transformation concatenates the input images into a resulting super image that crops based on the top left corner and crop size. Another way to implement a Mosaic transformation is defined in [68].

**Figure 13:**
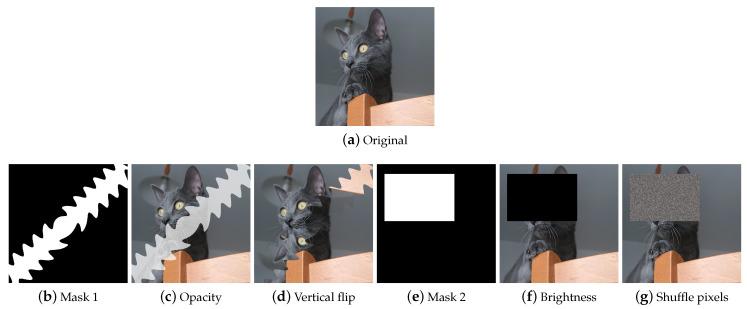
Data Augmentation by MaskedComposite. (**a**) the original input image. (**c**,**d**) are examples of unusual augments using mask (**b**). (**f**,**g**) show that by a square-shaped masking (**e**) and manipulating brightness or shuffling pixels, it is possible to replicate dropout.

**MaskedComposite** MaskedComposite (Figure 13) is a simple but powerful technique: given an image and a mask, it performs the user’s chosen operation only on the masked image areas; this means that it can also be used to replicate techniques such as dropouts or make more complex augments.

**Figure 14:**
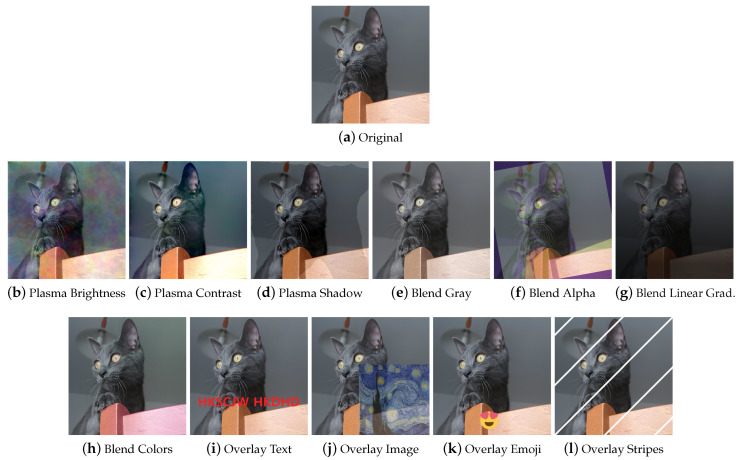
Example transformations based on plasma fractals, blend, and overlay transformations.

**Plasma Fractals** Plasma Fractals [62,69] (Figure 14b–d) are mostly used in computer graphics to simulate natural phenomena such as clouds or terrain. In addition to being used to generate textures and height maps, they can be employed in DA to achieve particular effects different from those obtained with traditional augmentation techniques.

**Blend** The Blend category represents those techniques that involve the complete fusion of two images. The second image can be different to or a variant of the first one, as shown in Figure 14e–h.

**Overlay** Depending on how they are used, overlay methods can look like MSDA or dropout. They are also used to add elements such as text or portions of images to images, to simulate memes and online content, or to specifically augment images such as in the libraries Automold (to add a layer of weather to the image) and Augraphy (for example, to add fake bindings to a document image). Some examples are shown in Figure 14i–l.

### 4.2. Task-Driven Traditional Techniques

**Figure 15:**
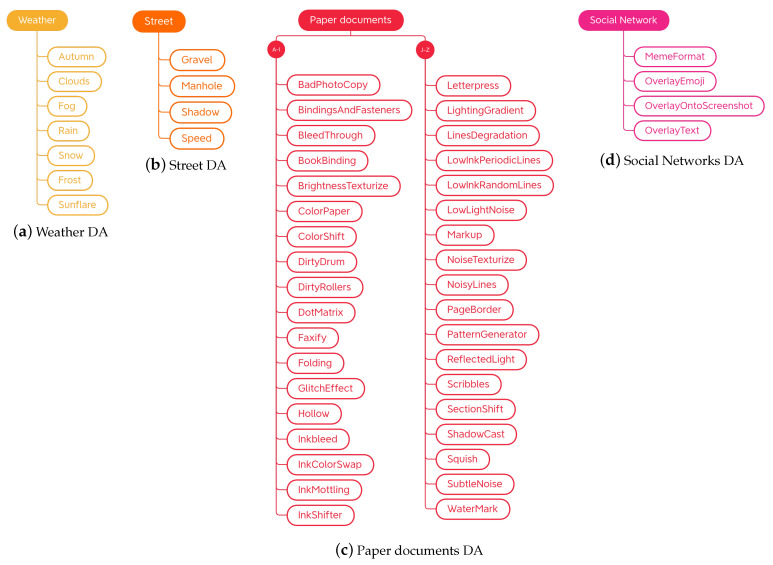
Taxonomy of the task-driven traditional data augmentation techniques.

Placed in *task-driven* traditional techniques are those techniques aimed at increasing the images in a certain domain, regardless of their functioning. During the analysis of the libraries, four groups of task-driven techniques were identified: methods for emulating certain atmospheric conditions, transformations for modifying street images, techniques dedicated to paper documents, and finally, transformations dedicated to the world of social networks. Subcategories of the task-driven traditional techniques and their organization are shown in Figure 15.

#### 4.2.1. Weather

This category groups all the transformations that change images in terms of atmospheric conditions. These techniques can find applications in the field of autonomous driving and outdoor video surveillance, as it is widely acknowledged that adverse weather conditions compromise the quality of acquired images and negatively impact the performance of algorithms that rely on such images [70]. The techniques that will be described below allow the user to create large datasets by which to train robust models for the cases mentioned above. Weather transformations are shown in Figure 15a.

**Figure 16:**
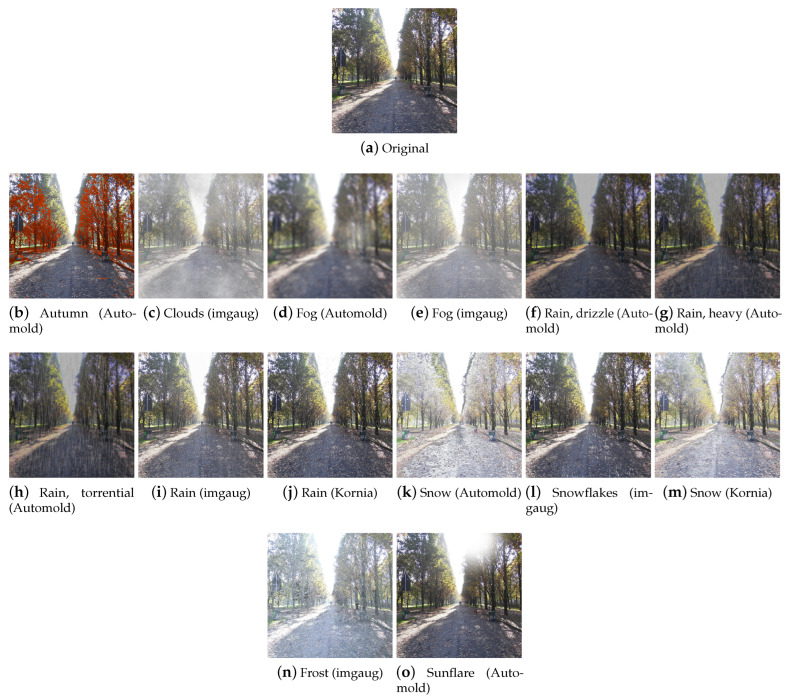
Effects of various task-driven weather transformations on a sample input image.

**Autumn** This technique adds autumnal colors to the input image. In the Automold library, this effect (Figure 16b) is obtained by replacing a randomly chosen autumnal color from a predefined list of four colors to pixels that meet the following conditions: the luminosity value of the HLS color space is between 20 and 100 (excludes regions that are too bright or dark) and the average of the values of the saturation channel in the considered area is less than 100 (small saturated areas are taken into consideration). The autumnal color is then assigned to the hue channel, setting the saturation of the modified pixels to the maximum value of 255.

**Clouds** This technique adds clouds to the image. It is available in imgaug, wherein, the CloudLayer class produces a single layer of clouds, while the Clouds class (Figure 16c) is a wrapper around CloudLayer that makes 1 to 2 layers per image, leading to variable densities and frequency patterns of the clouds.

**Fog** This technique simulates fog in the image. In Automold (Figure 16d) (and consequently in Albumentations, since it includes the Automold technique), the effect is achieved by blurring the image in random areas. In imgaug (Figure 16e), however, the Fog class is a wrapper around CloudLayer (described in the previous paragraph) that runs a single layer per image with a configuration that leads to quite dense clouds with low frequency patterns.

**Rain** This technique adds raindrops to the image. In Automold and Albumentations, the drops are drawn on the image one by one, after calculating the coordinates on which to place them. By setting the parameters, it is possible to decide the angle, the size, the color, and the type of rain, which can be chosen to be either drizzle (Figure 16f), heavy (Figure 16g), or torrential (Figure 16h). After drawing the drops, a blur effect is applied to give the rain a more realistic look. The imgaug library includes the RainLayer and Rain classes for adding rain. RainLayer adds a single layer of raindrops to images; it has several parameters that control the characteristics of the raindrops, such as their density, size, uniformity, angle, speed, and blur. The class also has several methods that are used internally to apply effects to images with specified parameters. For example, the _blend method mixes the original image with the noise of raindrops to create a more natural final effect. The Rain class (Figure 16i) is a wrapper around RainLayer and adds up to three raindrop layers to images. Furthermore, in Kornia (Figure 16j), the rain is drawn on the image drop by drop. It is possible to decide the number of drops and their size.

**Snow** When it comes to creating a snowy effect, there are two approaches: adding snowflakes one by one by drawing them on the image, or changing the brightness pixel values to create the effect of a snowy landscape.

Automold’s add_snow method (Figure 16k) (and its counterpart in Albumentations) creates the illusion of snow by whitening some pixels of the image: the brightness channel values (HLS color space) are scaled upwards by multiplying them by a brightness coefficient. Pixels with a value greater than 255 are clipped to 255. The image is then converted back to RGB.

The imgaug library offers both approaches. The SnowflakesLayer class generates a random noise pattern to represent snowflakes and adjusts their density, uniformity, size, motion blur angle, and perceptual falling speed; applies a Gaussian blur and motion blur to simulate the movement of snowflakes; and finally, combines the generated pattern with the input image. Snowflakes (Figure 16l) is a wrapper around SnowflakesLayer and is used to apply up to three layers of snow on the image. Snow is another class of imgaug with which it is possible to apply a snow effect to images by drawing snowflakes and is part of the imgcorruptlike module. Similar to Automold’s add_snow, FastSnowyLandscape simulates snow in the image by increasing brightness channel values in HLS space. Kornia’s RandomSnow (Figure 16m) also emulates snow by manipulating the brightness channel.

**Frost** Frost is a type of corruption that adds a frost or ice effect to images. The technique is available in imgaug (Figure 16n) and is part of the imgcorruptlike module.

**Sunflare** This technique adds sunflares to the image. The method present in Automold (Figure 16o) (and in Albumentations) creates a circular flare by superimposing concentric circles of different sizes and opacities on the original image, calculates the coordinates of the lines of light extending from the center of the sun, and adds random circular flares along these lines of light. The position and opacity of the flares are generated randomly.

#### 4.2.2. Street

The Automold library is the only library to provide techniques strictly dedicated to the modification of road images. Albumentations borrows two of these augments, add_gravel and add_shadow, renaming them RandomGravel and RandomShadow, respectively. The taxonomy for street-related data augmentations is organized as shown in Figure 15b.

**Figure 17:**
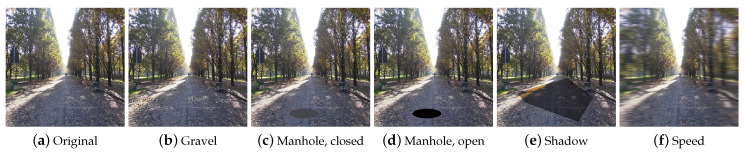
Effects of various task-driven street transformations on an example input image.

**Gravel** This technique simulates the presence of gravel on the road. The add_gravel augmenter (Figure 17b) generates a list of random coordinates representing the locations of the gravel patches in the image. Each gravel rectangle is created by operating on the values of the luminosity channel: by iterating on the rectangle, small patches of different sizes and luminosities are added that give the rectangle an appearance reminiscent of that of gravel.

**Manhole** The add_manhole technique draws an elliptical shape representing a manhole and overlays it on the original image. It is possible to choose the type of manhole between open and closed, which differ in color: if the manhole is closed (Figure 17c), the default color will be (67, 70, 75); while, if the manhole is open (Figure 17d), the color will be (0, 0, 0).

**Shadow** This technique adds shapes that emulate the shadows cast by buildings or other objects that come between the sun and the street. The add_shadow augmenter (Figure 17e) takes as input the desired number of shadows, their size, and the region of interest where the shadows will be drawn and generates a list of vertices for each shadow. Using this information, a mask is generated and applied to the original image by decreasing the brightness of the masked pixels.

**Speed** The add_speed function (Figure 17f) uses motion blur by applying it gradually from the left and right sides of the image towards the center to simulate movement.

#### 4.2.3. Paper Documents

**Figure 18:**
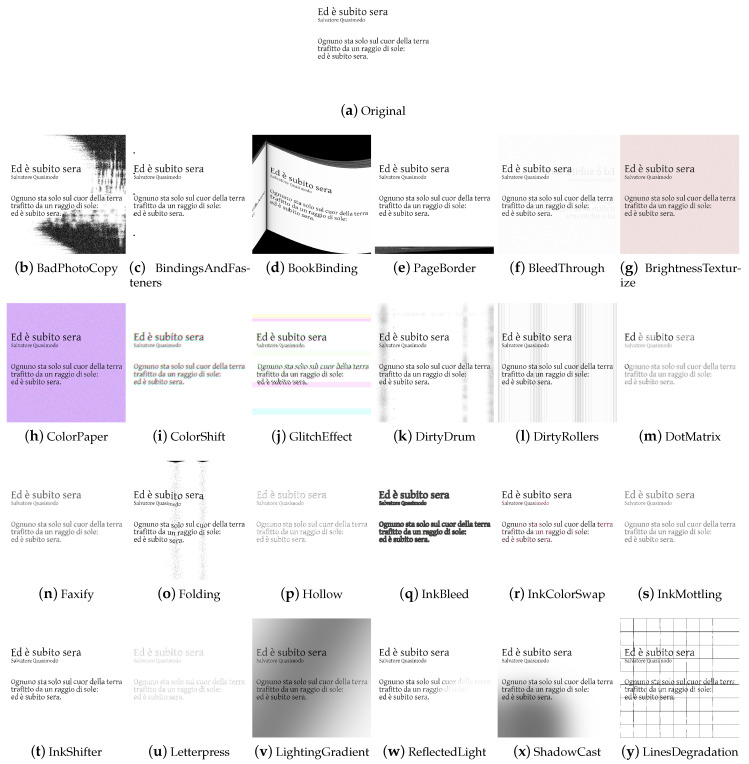
Some examples of techniques for simulating paper documents.

**Figure 19:**
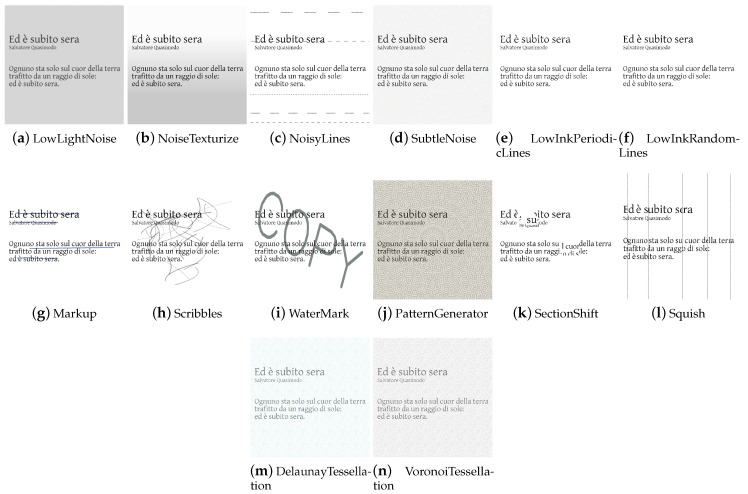
Other examples of techniques for simulating paper documents. The input image is depicted in Figure 18a.

Transformations dedicated to the production of synthetic paper documents (Figure 18 and Figure 19) were encountered only in the Augraphy library. As already anticipated in the description of the aforementioned library, starting from a clean image, degraded copies are obtained that, for example, seem to have been photocopied with a dirty photocopier, with scribbles, or handwriting. There are also techniques that modify the appearance of the paper, changing its color or simulating a certain texture. It also has to be noted that, while this section reports methods from a single library, these techniques may be implemented in the future by other libraries, and the Augraphy library also implements methods that are part of other groups of our taxonomy (e.g., geometry transformations).

The taxonomy of this category is organized as shown in Figure 15c. The entire list of these methods can be found not only on the site of the source library but also on the DALib site, which will be presented in Section 5. At the time of writing, some of the methods are implemented in the source code of the library but not yet available in its latest release.

**BadPhotoCopy** This technique adds noise to generate an effect of a dirty photocopier (Figure 18b). Four different types of noise can be introduced on the input image: even spread of noise, regular noise patterns, noise in all borders of the image, sparse and small noise.

**BindingsAndFasteners, BookBinding, PageBorder** These augmenters simulate the effects of bindings and borders as they appear in photocopied documents. Furthermore, Fasteners augmentation creates binder marks in the input image (Figure 18c). Binder marks are supported out-of-the-box (i.e., punch holes, binding holes, and clips) or can be user-supplied. BookBinding (Figure 18d) creates the effect of a page curling away from the scanner bed, towards the binding of a book or magazine. PageBorder (Figure 18e) creates an effect of a single or multiple page borders on any side of the page by stacking images multiple times.

**BleedThrough** The BleedThrough augmenter (Figure 18f) emulates the ink bleedthrough effect as the combination of ink bleed and Gaussian blur operations. The ink bleed is generated by using a random image or the reverse side of the provided image.

**BrightnessTexturize** BrightnessTexturize (Figure 18g) creates a random noise in the brightness channel of the clean input image to emulate paper textures.

**ColorPaper, ColorShift, GlitchEffect** The ColorPaper augmentation (Figure 18h) changes the color of the input paper based on user-provided hue and saturation. The ColorShift augmentation (Figure 18i) allows instead each color channel to be shifted by certain offsets to create a shifted color effect around the printed text. The GlitchEffect (Figure 18j) uses the ColorShift augmentation and also shifts patches of the image horizontally or vertically.

**DirtyDrum, DirtyRollers** The Dirty Drum and Dirty Rollers augmentations (Figure 18k,l) emulate deposits of dirt and ink-grime from dirty printers. Such defects appear as horizontal or vertical lines in the final image.

**DotMatrix** The DotMatrix augmentation (Figure 18m) creates a dot matrix effect by drawing dots of mean color in the detected contours of the printed characters. This allows the easy simulation of the effect of acquiring a digital image of a document printed using a dot matrix printer.

**Faxify** The Faxify augmenter (Figure 18n) introduces artifacts that emulate those created when faxing a document. Characters usually appears in a lighter ink color and with blurred edges.

**Folding** The Folding augmentation (Figure 18o) emulates the scanning of folded paper sheets by applying perspective transformations on portions of the page. It also introduces a visible warp effect around the fold lines.

**Hollow, InkBleed, InkColorSwap, InkMottling, InkShifter** These augmentation techniques deal with different effects that commonly affect ink-printed documents. The Hollow augmentation (Figure 18p) creates a hollow effect by replacing detected characters with their edges only. The detected contours are removed by using median filter operation. InkBleed (Figure 18q) uses the Sobel operator to create a mask of all edges, then applies random noise to those edges to simulate fuzzy edges typical of the ink bleed effect. The InkColorSwap (Figure 18r) augmentation replaces the color detected ink contours with a user-provided one. The InkMottling (Figure 18s) augmenter creates a random pattern effect on the detected text by blending it with a layer of random Gaussian noise. Finally, InkShifter (Figure 18t) applies a noise map on the image and displaces the text, thus, changing its morphology.

**Letterpress** The Letterpress augmenter (Figure 18u) produces regions of ink mimicking the effect of ink pressed unevenly onto the paper sheet. This allows the simulation of the effect of diverse ink retention when different pressures are applied while printing with letterpress or letterpunches.

**LightingGradient, ReflectedLight, ShadowCast** These techniques emulate the effect of lights and shadows on paper documents. Lighting Gradient (Figure 18v) produces a decayed light mask generated by a light strip on a given position and direction, and applies it to the image as a lighting or brightness gradient. The ReflectedLight augmentation (Figure 18w) creates a reflected light effect by drawing ellipses of different brightness, allowing the replication of the effect of image acquisition under direct lighting with strong reflections as on coated paper. Finally, the ShadowCast augmenter (Figure 18x) applies a shadow effect onto a portion of the image.

**LinesDegradation** LinesDegradation (Figure 18y) degrades vertical and horizontal lines by replacing them with lines formed by image gradients with a different values. As a result, the new image presents lines that have different thicknesses at different locations.

**LowLightNoise, NoiseTexturize, NoisyLines, SubtleNoise** These augmentations simulate the presence of different types of noise in the images. LowLightNoise (Figure 19a) simulates noise introduced due to low-light conditions. NoiseTexturize (Figure 19b) creates a random noise pattern to emulate paper textures. NoisyLines (Figure 19c) introduces noisy lines by drawing horizontal or vertical lines at fixed intervals. SubtleNoise (Figure 19d) emulates the imperfections in scanning solid colors due to subtle lighting differences.

**LowInkPeriodicLines, LowInkRandomLines** LowInkPeriodicLines and LowInk RandomLines (Figure 19e,f) create a set of lines that repeat in a periodic or random fashion, respectively, throughout the image. The effect is applied on text lines and emulates defects introduced in the print by a printer with low ink, dirty nozzles or an uncalibrated paper feed.

**Markup, Scribbles, WaterMark** These methods allow the simulation of the presence of handwritten marks, notes, highlights, and watermarks. The Markup augmentation (Figure 19g) uses contour detection to detect text lines and add a smooth text strikethrough, highlight, or underline effect. The intensity and color of these effects can be customized. The Scribbles augmentation (Figure 19h) applies random scribbles to image; differently from Markup, the scribbles are not constrained to text or lines in the input image. The WaterMark augmentation (Figure 19i) adds a watermark effect to the input image based on user input, which can be provided as a custom text or an image to be superimposed.

**PatternGenerator** The PatternGenerator (Figure 19j) creates aperiodic crystals and superimposes them onto the input image. This technique is based on the QuasiPattern Distortion augmentation techniques. The pattern can be controlled through its frequency and rotation, making it more or less frequent and symmetrical.

**SectionShift** The SectionShift augmenter (Figure 19k) shifts a single or multiple sections of the input image in the horizontal, vertical, or both directions, creating an effect of shifted image sections, while moving sections, parts of the image will be overwritten and the new section can be filled with random or user-provided color.

**Squish** The Squish augmentation (Figure 19l) creates a squish effect by removing a fixed horizontal or vertical section of the image. This results in portions of missing text and black lines inserted in the picture simulating glitches in the movement of the printing or scanning mechanism.

#### 4.2.4. Social Network

**Figure 20:**
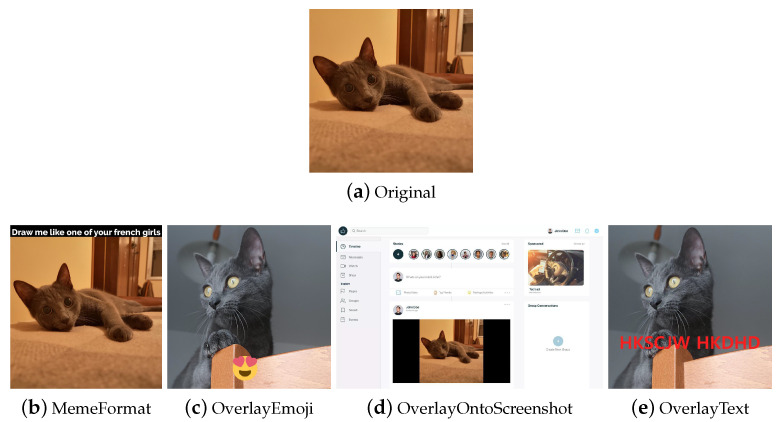
Examples of data augmentation techniques related to social networks.

The techniques dedicated to the production of images related to social networks (Figure 20) are provided only by the AugLy library. The methods described below make it possible to enrich the dataset with images that are similar to those produced by users’ activity on social networks.

**MemeFormat** (Figure 20b) formats an image to make it look like a meme, by adding text in a specific font and with a certain text and background color. **OverlayEmoji** (Figure 20c) overlays an emoji on an image, allowing the user to choose the position and size of the emoji. **OverlayOntoScreenshot** (Figure 20d) overlays the image onto a screenshot template. **OverlayText** (Figure 20e) superimposes text on the image and allows the user to specify its position, size, color, opacity, and font.

### 4.3. Generative Deep Learning

**Figure 21:**
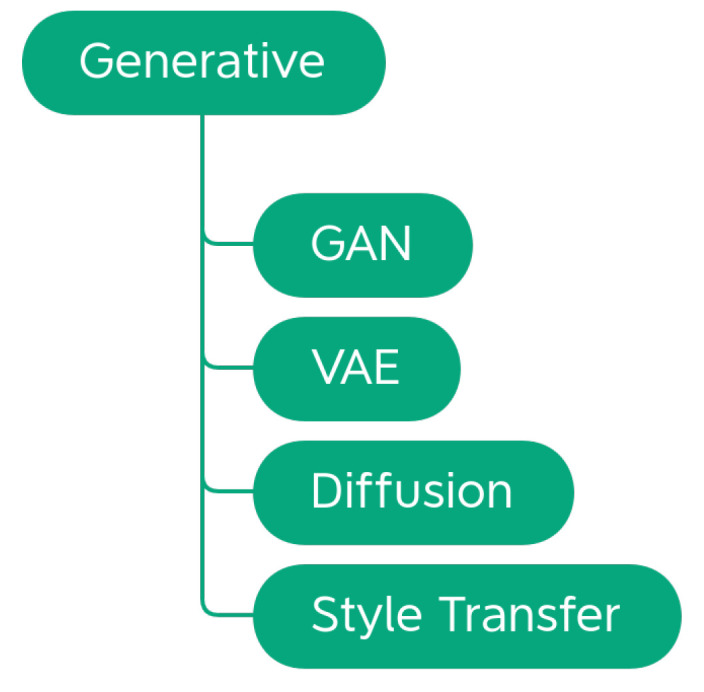
Taxonomy of the Generative Deep Learning data augmentation techniques class.

Generative Deep Learning algorithms (Figure 21) are a class of Machine Learning algorithms based on deep neural networks that have the ability to generate new realistic data similar to those present in the training dataset. Generative Neural Networks use several architectures, including Adversarial Generative Neural Networks (GANs) and Variational Autoencoder generative models (VAEs). The main goal of these networks is to learn the probability distribution of the training data so that new examples can be generated that are similar to the original ones. Even though no deep learning techniques were encountered during the analysis of the libraries, they will be described below for completeness.

#### 4.3.1. GANs

**Figure 22:**
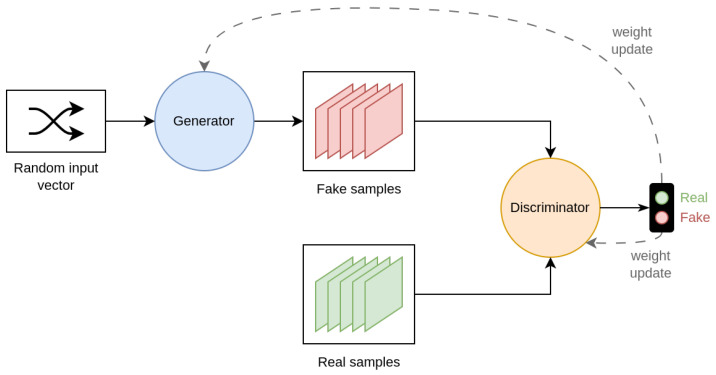
Graphical representation of the functioning of a GAN.

Generative Adversarial Networks (GANs) [71] are a type of machine learning algorithm that use two Neural Networks (NNs) to generate new images (Figure 22). The two NNs are a generator and a discriminator, respectively. The generator takes a random noise vector as input and is trained to generate images as similar as possible to the training set; the discriminator is trained to recognize real images from synthetic ones. The two networks are trained in an adversarial way, so that the generator produces increasingly realistic images while the discriminator is able to better and better distinguish the fake images from the real ones; at each iteration, the weights of the networks are modified based on the result of the discriminator.

#### 4.3.2. VAE

**Figure 23:**
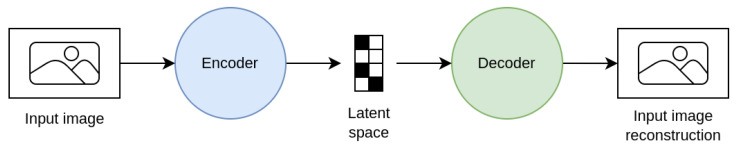
Graphical representation of how an autoencoder works.

Autoencoders (Figure 23) are Unsupervised Machine Learning models based on Artificial Neural Networks, which compresse an input and then subsequently reconstruct it. An Autoencoder consists of an encoder and a decoder; the encoder receives a certain data object as input and compresses it into a small vector called latent space; the decoder receives the vector of the latent space as input and tries to reconstruct the original image.

**Figure 24:**
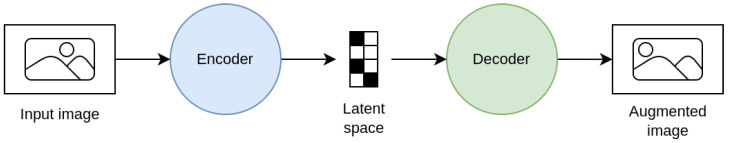
Graphical representation of how a VAE works.

Variational Autoencoders (VAEs) (Figure 24) are a type of autoencoder used for data augmentation. Unlike traditional autoencoders, VAEs are capable of generating new images by random sampling points within the latent space.

#### 4.3.3. Diffusion Models

**Figure 25:**
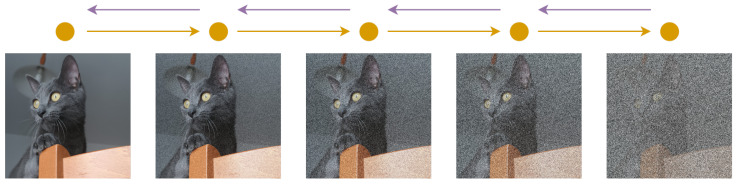
Graphical representation of the idea behind diffusion models.

Let us imagine having an image and systematically applying Gaussian noise to it (see Figure 25); at a certain point, we will have an image that is unrecognizable from the initial one and that will be composed only of noise. Let us now imagine carrying out the reverse process; is it possible, starting from an image of only noise, to arrive at the original image? This is the idea behind Diffusion Models. In a Diffusion Model, a new image is iteratively obtained through the diffusion process, i.e., the propagation of noise through the image. At each iteration, the noise is decreased and the image is modified to approach the probability distribution of the training dataset.

#### 4.3.4. Style Transfer

**Figure 26:**
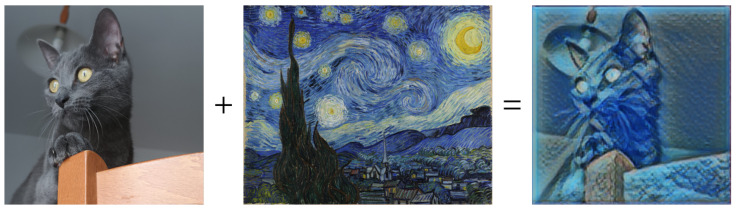
Graphical representation of the functioning of the Style Transfer.

Style Transfer (Figure 26) is a technique that, starting from two images, allows a new image to be obtained that has the content of the first and the style of the second. A NN extracts the feature maps of the input images and isolates the content and style information. Subsequently, the style information is used to modify the representation of the content image and, thus, obtain the augmented image.

## 5. The DALib Website

Some of the libraries analyzed, although they have good documentation, do not provide examples. DALib was born to have clear and immediate examples of the functioning of the methods of the major image augmentation libraries and to quickly disclose the primary information about them. We could define DALib as a catalog of Data Augmentation libraries for Image Processing.

DALib was produced using the Jekyll static site generator and is hosted on GitHub.

### 5.1. The Homepage

**Figure 27:**
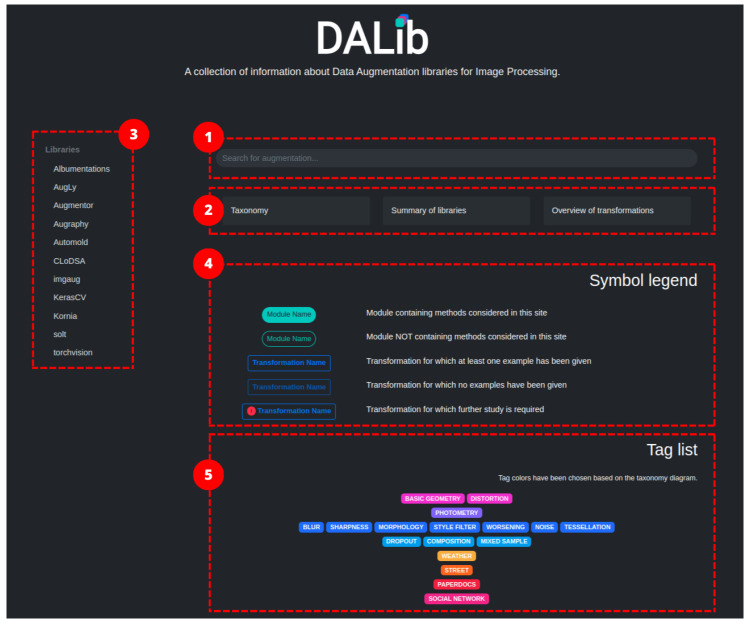
Screenshot of the DALib website homepage. The numbers, in order, highlight: (**1**) the search bar, (**2**) link buttons, (**3**) the library menu, (**4**) the legend of symbols used on the site, and (**5**) the list of tags related to methods.

The homepage (Figure 27) contains a search bar to look for an augment among the libraries present on the site, the list of which is fixed on the left side of the page. This list is a clickable menu that redirects to the site page related to the selected library. The homepage contains links to the method taxonomy, a summary of key library information, and a list of all transformations encountered; it also presents a legend of the symbols used on the site and the list of tags related to methods.

### 5.2. The Taxonomy Page

The taxonomy page consists of a navigable card with which it is possible to explore the taxonomy. The first tab shows the overall skeleton of the taxonomy; by selecting the following tabs, it is possible to receive more information on each section. Please refer to Section 3 for additional details about the taxonomy organization.

### 5.3. The Library Pages

**Figure 28:**
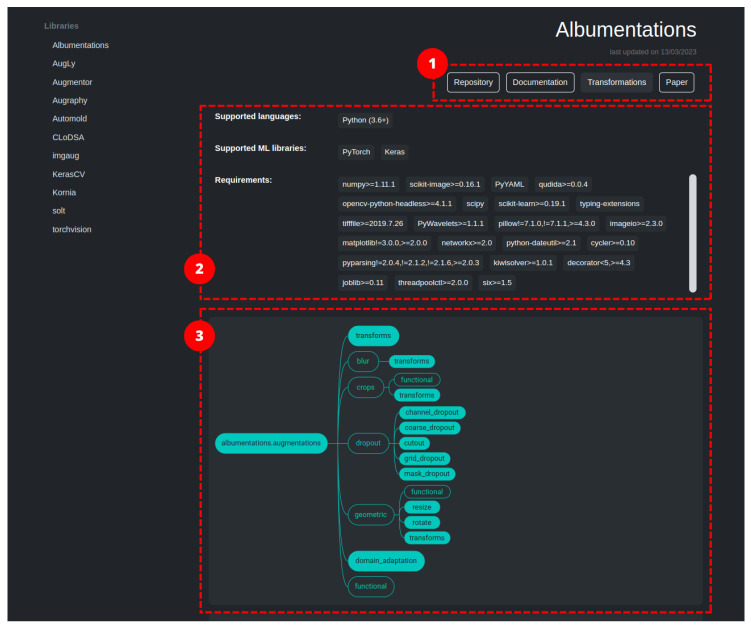
Screenshot of a DALib site page related to a library. The numbers, in order, highlight: (**1**) links to the repository, documentation, list of transformations, and articles, (**2**) information about supported languages and supported machine learning libraries and library requirements, and (**3**) module diagram.

The library pages (e.g., Figure 28) have buttons for links to the repository, documentation, list of transformations, and any related articles. Immediately following, there is a section containing more technical information that, when necessary, lists the supported machine learning languages and libraries, as well as the requirements of the library. For the libraries that provide dedicated data augmentation modules or packages, an explanatory logical diagram is also provided. This diagram indicates to which modules the methods listed in the transformation table belong.

**Figure 29:**
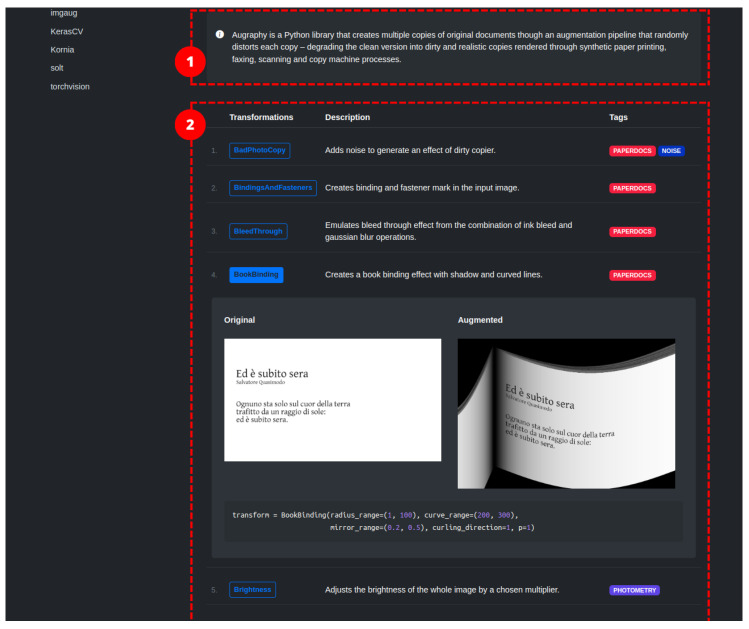
Screenshot of a DALib site page related to a library. The numbers, in order, highlight: (**1**) information box and (**2**) transformation table.

Before the table, there is a box with a brief introduction to the library (e.g., Figure 29). The transformation table contains the list of augmentations, with a description of the method and related tags. The tag colors are a reference to the colors used in the taxonomy diagram. As indicated in the previously mentioned legend, if the button is active (it has a brighter color than a disabled button), clicking on it will display the sample images (original and augmented), and a box with the code used to obtain the augmentation.

Note that the last update date reported immediately below the library name refers to the last update of the DALib page, and not the last update of the library.

### 5.4. A Few Use Cases

The DALib website presents an intuitive user interface and a linear organization of its contents. In addition, we describe in this section a few common use cases to guide the users in recovering the information for which they are searching.

Let us suppose that the user is approaching image augmentation for the first time: they will find the navigation of the taxonomy interesting and after having consulted the diagrams, they will have clearer ideas on the various types of augmentations. Since our user is not an expert on the subject, they feel the need to better understand the function of some techniques, so they return to the homepage and type, in the search bar, the name of the technique that they want to know more about; the result of the search shows the list of functions relating to the entered technique that are inside the libraries. For each function, the information provided is the name of the function, the name of the library to which it belongs, the name of the taxonomic class to which the reference technique belongs, and the beginning of the description relating to the method. At this point, the user can choose to select one of the items: they will be redirected to the library page corresponding to the selected method and be able to read the complete description of the method and, if available, take advantage of the images and example code.

Now, let us assume that the user has clear goals that they want to achieve by applying data augmentation to their dataset. They already know the techniques that they want to apply to the images but they want to know which library is the most suitable for their purposes; they, therefore, open the “Overview of transformations” page. The user now has the table in front of them; they can scroll the table to find the row relating to the technique concerned and will be able to exclude the libraries that do not display that technique, denoted by the appropriate icon. To get more information, the user can click on the filled icons: a popover will open containing the list of all the methods relating to the technique and the library in question. Each item is clickable and refers to the method on the page of the library it belongs to.

## 6. Challenges

Notwithstanding the advantages of data augmentation, some issues must be considered [72,73]. One of the primary concerns is the risk of overfitting [4]. When augmentation is applied excessively or inappropriately, the model may become overly specialized in recognizing the augmented patterns in the training data. This can lead to poor generalization to real-world, unseen data, which is a significant issue in machine learning. Additionally, data augmentation increases the computational load during training. By effectively expanding the training dataset, it necessitates longer training times and greater computational resources, which can be especially problematic when working with large datasets or complex models [74]. Another potential issue is the introduction of artifacts and noise into the data. If augmentation techniques are poorly chosen or overused, they can distort the original information in the data, negatively impacting the model’s ability to learn meaningful features.

In datasets with significant inherent variation, such as those from diverse sources or real-world scenarios, augmentation may provide limited benefits [2]. In such cases, applying augmentation excessively can even hinder model performance rather than improving it. Moreover, selecting appropriate augmentation strategies requires domain-specific expertise. Not all augmentation techniques are suitable for every problem domain, and making the wrong choices can diminish the effectiveness of data augmentation.

Data augmentation can also exacerbate class imbalance issues in a dataset [72,75]. If augmentation is not applied uniformly across different classes, it can lead to biased model predictions, which is especially problematic in tasks with imbalanced class distributions.

Furthermore, there are practical considerations, such as increased storage requirements for augmented data. This can be a concern when dealing with large datasets, multiple augmentation techniques, or limited storage resources [72]. The implementation and maintenance of augmentation pipelines can introduce complexity to the workflow leading to an increase in the computational cost during model training. Augmentation requires applying transformations to each training example during each training iteration, which can significantly increase the computational cost.

To address these potential drawbacks, practitioners must carefully select and fine-tune augmentation techniques based on the dataset’s characteristics and the specific problem domain. Regular monitoring of the model’s performance on a validation set is crucial to ensure that data augmentation is not causing overfitting or other issues. Experimentation with different augmentation strategies and levels can help strike the right balance between improving model robustness and avoiding these challenges.

## 7. Conclusions

This paper presented a comprehensive survey of publicly available data augmentation libraries. The objective was to assist practitioners in navigating through these libraries by offering a curated taxonomy of different approaches. The goal was not to identify the best data augmentation library, but rather to provide an overview of their content, enabling users to select the most suitable data augmentation strategies for their specific problems.

To achieve this, we extensively researched the available data augmentation libraries, with a specific focus on those relevant to computer vision tasks. We extracted and categorized the implemented methods from each library, creating a taxonomy of augmentation methods. We collected information on data augmentation libraries designed specifically for computer vision applications, bridging a gap in existing reviews that primarily focus on data augmentation methods rather than the availability of such methods in public libraries. Our survey is not limited to a particular application scenario but offers readers a comprehensive overview of available methods that can be utilized in different computer vision applications.

This paper is the first to provide a comprehensive review of data augmentation libraries while also presenting a curated taxonomy. The taxonomy, methods, and examples associated with the surveyed data augmentation libraries are gathered and accessible on a dedicated public website (DALib: http://www.ivl.disco.unimib.it/activities/dalib/, accessed on 10 October 2023). This website serves as a centralized repository for researchers and practitioners to navigate and consult the information. This work is an ongoing project, and we plan to expand it as new libraries become available. By continuously updating the survey and the DALib website, we aim to provide an up-to-date public resource in data augmentation methods for the computer vision community.

## Figures and Tables

**Table 1:** Data Augmentation libraries (links accessed on 10 October 2023).

Library Name	GitHub Repository	Last Update
Albumentations [21]	albumentations-team/albumentations (https://github.com/albumentations-team/albumentations)	2023
AugLy [22]	facebookresearch/AugLy (https://github.com/facebookresearch/AugLy)	2023
Augmentor [23]	mdbloice/Augmentor (https://github.com/mdbloice/Augmentor)	2023
Augraphy [24]	sparkfish/augraphy (https://github.com/sparkfish/augraphy)	2023
Automold [25]	UjjwalSaxena/Automold–Road-Augmentation-Library (https://github.com/UjjwalSaxena/Automold--Road-Augmentation-Library)	2022
CLoDSA [26]	joheras/CLoDSA (https://github.com/joheras/CLoDSA)	2021
imgaug [27]	aleju/imgaug (https://github.com/aleju/imgaug)	2020
KerasCV [28]	keras-team/keras-cv (https://github.com/keras-team/keras-cv)	2023
Kornia [29]	kornia/kornia (https://github.com/kornia/kornia)	2023
SOLT [30]	Oulu-IMEDS/solt (https://github.com/Oulu-IMEDS/solt)	2022
Torchvision [31]	pytorch/vision (https://github.com/pytorch/vision)	2023

**Table 2:** Overview of Data Augmentation libraries (stats at 10 October 2023). # indicates the number of data augmentation techniques in the library.

Library Name	Language	ML Frameworks	#	GitHub Stars	Citations	Referenced
Albumentations [21]	Python	PyTorch, Keras	83	12,600	1457	1540
AugLy [22]	Python	PyTorch	38	4800	33	117
Augmentor [23]	Python, Julia	PyTorch, Keras, Flux	23	5000	292	729
Augraphy [24]	Python	—	26/40	233	4	9
Automold [25]	Python	—	17	566	6	54
CLoDSA [26]	Python	Keras	26	119	65	86
imgaug [27]	Python	—	164	13,800	200	1460
KerasCV [28]	Python	Keras	21	838	—	39
Kornia [29]	Python	PyTorch	64	8700	233	645
SOLT [30]	Python	PyTorch	21	258	11	11
Torchvision [31]	Python, C++, Java	PyTorch	27	14,600	415	6770

**Table 3:** List of transformations in the analyzed libraries grouped by category. Albm: Albumentations; Agly: AugLy; Augm: Augmentor; Agpy: Augraphy; Amld: Automold; CLSA: CLoDSA; imga: imgaug; KsCV: KerasCV; Krna: Kornia; SOLT: SOLT; Trch: Torchvision. Transformations are grouped by category identified by colors: 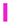 Geometry; 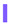 Photometry; 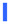 Quality; 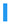 Miscellaneous; 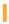 Weather; 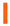 Street; 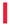 Paper documents; 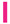 Social Network; 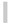 Other.

	Albm	Agly	Augm	Agpy	Amld	CLSA	Imga	KsCV	Krna	SOLT	Trch
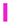 Resize ([35], Table 2.3)	√	√	√	√	.	√	√	√	√	√	√
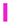 Rotate ([35], Table 2.3)	√	√	√	√	.	√	√	.	√	√	√
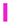 Translate ([35], Table 2.3)	√	.	.	√	.	√	√	.	√	√	√
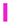 Shear ([35], Table 2.3)	√	√	√	.	.	√	√	√	√	√	√
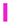 Flip ([35], Table 2.3)	√	√	√	√	√	√	√	.	√	√	√
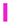 Crop	√	√	√	√	.	√	√	√	√	√	√
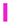 Pad	√	√	.	.	.	.	√	.	√	√	√
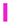 Perspective ([36], Section 4.1)	√	√	√	.	.	.	√	.	√	√	√
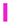 Zoom [23]	.	.	√	.	.	.	.	.	.	.	.
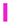 Transpose	√	.	.	.	.	.	.	.	.	.	.
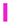 Distortion [37,38], ([39], Section 6.3)	√	.	√	.	.	√	√	.	√	.	.
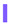 Hue ([35], Section 6.1)	√	.	√	.	.	√	√	√	√	√	√
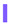 Saturation ([35], Section 6.1)	√	√	.	.	.	√	√	√	√	√	√
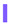 Brightness ([35], Section 6.1)	√	√	√	√	√	√	√	.	√	√	√
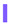 Grayscale [40,41,42]	√	√	√	.	.	.	√	√	√	.	√
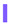 Invert ([35], Section 3.2)	√	.	√	.	.	√	√	.	√	.	√
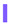 Sepia [43]	√	.	.	.	.	.	.	.	√	.	.
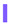 Solarize ([44], Chapter 4)	√	.	.	.	.	.	√	√	√	.	√
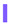 IntensityRemap [45]	.	.	.	.	.	.	.	.	.	√	.
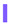 Contrast ([35], Section 3.2)	√	√	√	√	.	√	√	√	√	√	√
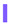 Color [21,26,27,28,29,46,47]	√	.	.	.	.	√	√	√	√	.	.
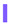 Opacity [22]	.	√	.	.	.	.	.	.	.	.	.
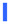 Blur ([35], Sections 3.5 and 6.6)	√	√	.	.	.	√	√	.	√	√	√
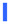 Noise ([35], Sections 5.2 and 6.8)	√	√	.	.	.	√	√	.	√	√	.
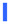 Compression ([35], Chapter 8)	√	√	.	√	.	.	√	.	.	√	.
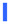 Sharpness ([35], Sections 3.6 and 6.6)	√	√	.	.	.	√	√	√	√	.	√
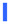 Math. Morphology ([35], Sections 9.2 and 9.3)	.	.	.	.	.	.	.	.	√	.	.
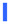 Posterization ([44], Chapter 3)	√	.	.	.	.	.	√	√	√	.	√
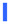 Cartoon [27]	.	.	.	.	.	.	√	.	.	.	.
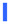 Tessellation [48,49]	√	√	.	.	.	.	√	.	.	.	.
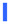 RingingOvershoot [50]	√	.	.	.	.	.	.	.	.	.	.
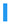 RandomErasing [51]	.	.	√	.	.	.	.	.	√	.	√
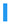 GridMask [52]	√	.	.	.	.	.	.	√	.	√	.
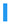 CutOut [53]	√	.	.	.	.	.	√	√	.	√	.
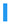 MaskDropout [54]	√	.	.	.	.	.	.	.	.	.	.
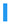 PixelDropout	√	.	.	.	.	√	√	.	.	.	.
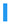 AugMix [55]	.	.	.	.	.	.	.	√	.	.	√
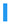 MixUp [56,57]	.	.	.	.	.	.	.	√	√	.	.
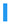 CutMix [58]	.	.	.	.	.	.	.	√	√	.	.
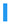 FourierMix [59]	.	.	.	.	.	.	.	√	.	.	.
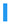 Mosaic [29]	.	.	.	.	.	.	.	.	√	.	.
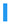 Jigsaw [60]	√	.	.	.	.	.	√	.	√	.	.
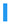 Overlay [22]	.	√	.	.	.	.	.	.	.	.	.
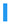 Blend [27,61]	√	.	.	.	.	.	√	.	√	.	.
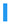 MaskedComposite [22]	.	√	.	.	.	.	.	.	.	.	.
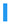 Plasma Fractals [62]	.	.	.	.	.	.	.	.	√	.	.
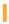 Autumn [25]	√	.	.	.	√	.	.	.	.	.	.
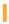 Cloud [27]	.	.	.	.	.	.	√	.	.	.	.
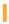 Fog [25,27]	.	.	.	.	√	.	√	.	.	.	.
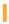 Rain [25,27]	√	.	.	.	√	.	√	.	√	.	.
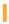 Snow [25,27]	√	.	.	.	√	.	√	.	√	.	.
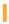 Frost [27]	.	.	.	.	.	.	√	.	.	.	.
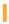 Sunflare [25]	√	.	.	.	√	.	.	.	.	.	.
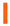 Gravel [25]	√	.	.	.	√	.	.	.	.	.	.
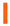 Manhole [25]	.	.	.	.	√	.	.	.	.	.	.
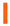 Shadow [25]	√	.	.	.	√	.	.	.	.	.	.
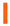 Speed [25]	.	.	.	.	√	.	.	.	.	.	.
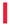 Paper documents [24]	.	.	.	√	.	.	.	.	.	.	.
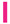 Social [22]	.	√	.	.	.	.	.	.	.	.	.
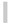 AutoAugment [34]	.	.	.	.	.	.	.	.	√	.	√
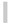 RandAugment [63]	.	.	.	.	.	.	√	√	√	.	√
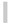 TrivialAugmentWide [64]	.	.	.	.	.	.	.	.	√	.	√

## Data Availability

The libraries surveyed in this paper are available at the following links (all accessed on 10 October 2023): Albumentations—https://github.com/albumentations-team/albumentations, AuLy—https://github.com/facebookresearch/AugLy, Augmentor—https://github.com/mdbloice/Augmentor, augraphy—https://github.com/sparkfish/augraphy, Automold—https://github.com/UjjwalSaxena/Automold--Road-Augmentation-Library, CLoDSA—https://github.com/joheras/CLoDSA, imgaug—https://github.com/aleju/imgaug, KerasCV—https://github.com/keras-team/keras-cv, Kornia—https://github.com/kornia/kornia, SOLT—https://github.com/Oulu-IMEDS/solt, torchvision—https://github.com/pytorch/vision. Results obtained by this survey are also available at http://www.ivl.disco.unimib.it/activities/dalib.
